# Mutations overlying the miR172 target site of TOE-type genes are prime candidate variants for the double-flower trait in mei

**DOI:** 10.1038/s41598-024-57589-8

**Published:** 2024-03-27

**Authors:** Stefano Gattolin, Elisa Calastri, Maria Rosaria Tassone, Marco Cirilli

**Affiliations:** 1 Institute of Agricultural Biology and Biotechnology (IBBA), CNR - National Research Council of Italy, 20133 Milan, Italy; 2 Department of Agricultural and Environmental Sciences (DISAA), University of Milan, 20133 Milan, Italy; 3 CREA Research Centre for Genomics and Bioinformatics, Via Paullese 28, 26836 Montanaso Lombardo , LO Italy

**Keywords:** Genetics, Plant sciences

## Abstract

Mutations affecting flower shape in many plants have been favored by human selection, and various fruit trees are also grown for ornamental purposes. Mei (*Prunus mume*) is a dual purpose tree originated in China well known in the Western world for its generous early blooms, often bearing double flowers. Building on the knowledge of its genomic location, a candidate gene approach was used to identify a 49 bp deletion encompassing the miR172 target site of the euAP2 gene *pmTOE* (*PmuVar_Ch1_3490*) as a prime variant linked to flower doubleness. Searching within a large dataset of genome sequencing data from Eastern germplasm collections demonstrated a tight variant-trait association, further confirmed in a panel of commercial and non-commercial varieties available in Italy. Moreover, two SNP mutations in the miR172 target site of *pmPET* (*PmuVar_Ch1_1333*) were identified in some double flower accessions. The mei orthologue of PETALOSA genes already found responsible for the phenotype in other plants suggests that independent variants may have been selected throughout mei domestication history.

## Introduction

Mei (*Prunus mume* Sieb. et Zucc.) is a tree species native to southwest China, where it grows as an elegant small to medium sized tree, highly appreciated for its flamboyant and fragrant bloom. Evidence for mei drupes collected for food and offerings traces back to the Neolithic age, about 7,500 years ago^1^, being still nowadays an important double-purpose (food and ornamental) crop in some Far East countries. Mei apricot-like fruits are harvested in mid to late summer, and processed into different food products ranging from preserves, jam, syrup, wine and vinegar^2^. In some environments, mei trees bloom in the coldest months of the year (from early December until March in the winter season), being a symbol of resilience, vitality and dignity, praised in countless poems and depicted in paintings and crafts throughout Chinese history. The ornamental value of mei trees was appreciated as early as the third century; mei trees producing flowers of various color, size and types (either single and double), were documented during the Tang Dynasty ruling from the seventh to the tenth century, when they were also introduced to Japan^2^ and referred to as Ume.

The classification of the mei germplasm is rather complex and cultivars are generally divided into various groups (Cinnabar Purple, Pendulous, Green Calyx, Versicolor, Apricot Mei, Meiren Mei, Pink Double, Albo-plena, Flavescent, Tortuosa and Single Flowered), sometimes without an univocal identification of independent genotypes^3^. In the Western countries, *P. mume* is still widely and often erroneously referred to as Chinese Plum or Japanese Apricot. Many Japanese varieties became available in European countries after the Paris Exposition Universelle in 1878^4^, although the original Japanese names of the cultivars were not maintained, resulting in their spread with new denominations, such as ‘Alba’, 'Alboplena', ‘Pendula’ or ‘Alphandii’; many varieties available in Europe and US are therefore likely synonymous with the true Japanese and Chinese counterparts.

The completion of *P. mume* whole-genome reference, along with the availability of genome sequencing data of wide Eastern germplasm collections^5–8^, is allowing the investigation of the genetic basis of important traits, such as cold resistance, plant architecture, flower type, color and aroma, as well as the development of useful tools for molecular breeding in this species. Flowering mei represent a prime choice for landscaping, due to its extreme blooming precociousness and traits associated to flower appearance, such as petal color and flower shape. The number of petals is an utmost important character determining the overall flower shape and size. Based on corolla diameter, mei flowers have been classified into shallow dish-shaped single flowers (hereafter SF) with 5 (or rarely 6) petals vs. bowl-shaped double flowers (DF), with a variable number of extra-petals ranging from 15 to over 25^9^.

Elucidating the genetic basis underlying flower size diversity is instrumental for deepening our knowledge on *Prunus* flower biology and to assist breeding for the ornamental market. Nevertheless, a variant and/or gene responsible for single or double flower type has remained elusive in mei. Recent QTL mapping experiments support a relatively simple inheritance of the trait, identifying a major locus on chromosome 1 affecting flower diameter^8,10^, although the large mapping interval hindered a clear positioning and identification of the causal mutation(s) or candidate gene variant(s). By contrast, several studies in Rosaceae and other plant families has shed light on different classes of mutations leading to the double flower phenotype^11–16^. In these species, the trait was linked to mutations in the euAP2-mir172 module integrated within the ABCDE model proposed to explain flower development: the repression of translation of an *euAP2* gene by miR172 ensures organ determinacy and the definition of the boundary between the reproductive inner flower parts and the outer sterile perianth^17–19^. In peach, a species closely related to mei, a recessive double-flower phenotype was linked to disruptions in the gene encoding *mir172d*, while a deletion depriving *Prupe.6G242400*, a TOE-like euAP2 gene, of its miR172 recognition sequence was proposed as the causal mutation in plants where the trait had dominant inheritance. Support for the crucial role of such mutations was further found in rose species, carnation and petunia, where analogous variants were identified in the corresponding orthologous TOE-like genes, all belonging to a subclade named PETALOSA^13^.

Building on the knowledge of the genomic position of the major-linked QTL controlling flower shape in mei, the availability of wide genomic and phenotypic information in mei germplasms and the conservation of genetic mechanisms across phylogenetically distant Eudicots, in this study the genetic determinant of the dominant DF trait in *P. mume* was investigated. Comparative genomic analysis within the map interval allowed to identify a 49 bp deletion in a TOE-type (*pmTOE*) gene closely related to PETALOSA (*pmPET*), partially ablating the miR172 recognition site. Sequencing-based genotyping of mei accessions from Eastern germplasm collections confirm the strict association of this *pmTOE*^*DEL*^ allelic variant with flower shape, and allowed to identify likely alternative causative variants in two independent SNPs in the miR172 recognition sequence of *pmPET* (*pmPET*^*SNP1*^*, pmPET*^*SNP2*^). Finally, molecular analysis of European commercial mei varieties, confirmed the association of *pmTOE*^*DEL*^ with flower shape.

## Results

### Identification of a candidate DF variant in *PmTOE*, a TOE-type euAP2 gene

The dissection of the genetic architecture of DF trait in mei was recently reported in three independent studies, using accessions panels and/or bi-parental cross-progenies. Using a wide panel of wild and cultivated mei accessions from different regions of China, major signals associated to petals number were identified in a region spanning ~ 3.7 Mb (4,058,003–7,693,997) on chromosome Pa1^6^. However, above-threshold signals were also present within the unanchored scaffolds. In an F_1_ ‘Liuban’ (SF) × ‘Huang Lve’ (DF) mei progeny using floral diameter as an estimator of flower doubleness, strongly associated markers were mapped in a 5 Mb interval on Pa1, with signal peaks covering the interval between 4,580,444 and 6,253,182 bp^8^, but no information was provided about the type of DF trait inheritance. Segregation of the DF trait was analyzed in another F_1_ progeny derived from the cross of mei accessions ‘XM’(SF) × ‘FP’(DF), showing a segregation ratio SF vs DF of about 1:1^10^ consistent with a dominant inheritance of the causative variant(s). According to the position of the main BSA-seq peaks interval detected on Pa1 (spanning a large region roughly comprised between 3.0 and 14.0 Mbp) a series of molecular markers were developed by the authors, showing an accuracy (for some of them) close to 100% in a panel of 69 mei SF and DF cultivars^10^.

**Figure 1:**
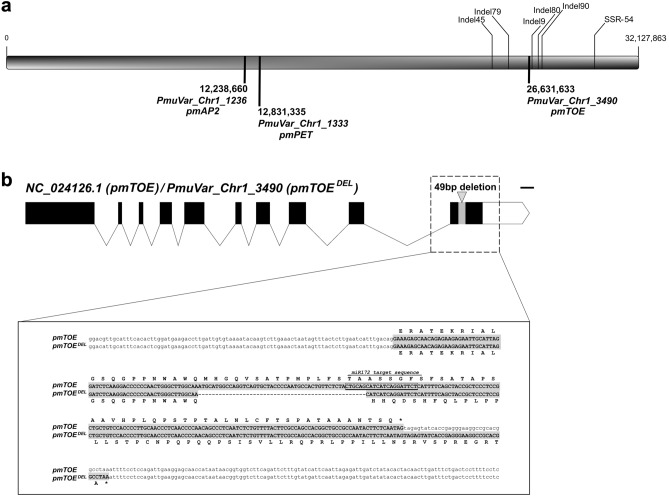
*pmTOE* chromosome location and *pmTOE*^*DEL*^ allele. (**a**) Schematic representation of *P. mume var. tortuosa* chromosome 1 with the position of *pmTOE* and genes encoding two other euAP2 transcription factors (pmAP2 and pmPET). Map position of markers previously reported as associated to the DF phenotype^10^ is indicated. (**b**) Schematic representation of the *pmTOE* gene structure with coding exons as black boxes (bar = 100 bp), the 49 bp deletion in *pmTOE*^*DEL*^ encompasses the miR172 target sequence and causes a frameshift of the coding sequence, as shown in more detail as sequence comparison of the gene region where the miR172 target sequence of *pmTOE* is boxed.

The amplitude of the associated intervals in these studies hinders the search for candidate genes. However, the dominant inheritance of mei DF trait (at least in the ‘XM’ × ‘FP’ progeny) and the strong conservation of *miR172-AP2/TOE* module across species was suggestive of an involvement of mei euAP2 genes, possibly caused by a mutation affecting the miR172 target site within^13^. In order to test this hypothesis, all associated markers^10^ previously mapped on the P.mume_V1.0 genome reference (from a wild *P. mume* species collected in Tibet, accession No. BJFU1210120008)^5^ were re-mapped on the recently released double-flowered *P. mume var. tortuosa* genome^7^. In this improved genome version, the Pa1 chromosome organization was rebuilt, allowing to correctly repositioning almost all the associated markers (including the unanchored ones) within the interval between 24.7 and 32.1 Mb (Fig. 1). Interestingly, this region hosts only one gene sequence encoding an euAP2 transcription factor, annotated as *PmuVar_Ch1_3490* (Chr1: 26,631,633 to 26,635,047) and belonging to the TOE-like family (hereafter named *PmTOE*). Outside the mapped interval, two additional euAP2 genes sequences were found, annotated as *PmuVar_Ch1_1333* and *PmuVar_Ch1_1236*. Blast search of *PmTOE* confirmed that the gene (NCBI code NC_024126.1) was originally mapped to the Ch1: 6,036,293–6,040,539 position in *P. mume* V1.0 assembly, in accordance with previous works, providing a first evidence of the involvement of this euAP2 gene in the DF trait.

Considering that wild mei accessions from Tibet^20^ and *P. mume var. tortuosa* bear single and double-flowers, respectively, genomic sequences from the two reference assemblies were aligned and compared with those of peach and apricot (Supplementary File 1A), allowing to identify a major variant between the wild *P. mume* and *P. mume var. tortuosa*, consisting in a 49 bp deletion in the DF *tortuosa* allele, *PmuVar_Ch1_3490*, affecting the miR172 target site (Fig. 1). The DF-associated *tortuosa* allele was named *PmTOE*^*DEL*^, leaving the denomination *PmTOE* for the SF-associated allele from wild *P. mume* accessions (Supplementary File 1B), also more similar to the peach and apricot orthologs. Since mutations at the miR172 target site of euAP2 genes have previously reported to induce dominant DF phenotype in various species^13^, the *PmTOE*^*DEL*^ variant was considered a prime candidate for the trait.

### pmTOE belongs to a TOE-like euAP2 clade closely related to PETALOSA

**Figure 2:**
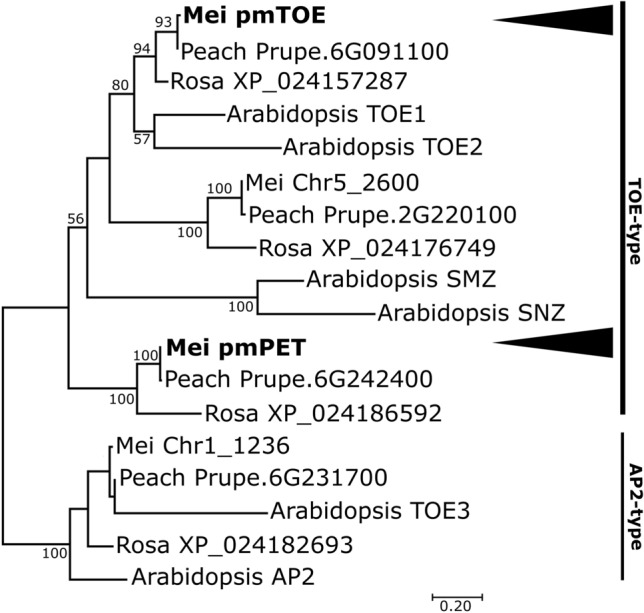
Evolutionary relationships among AP2-type and TOE-type euAP2 proteins in different plant species. The tree was obtained using the peptide sequences from *P. mume* (Mei), *P. persica* (Peach), *R. chinensis* (Rosa) and *A. thaliana* (Arabidopsis). Arrowheads indicate the position of *pmTOE* and *pmPET*.

A phylogenetic analysis was conducted to study the degree of relatedness between the AP2/TOE-like protein sequences and other euAP2 sequences in the mei genome and previously identified sequences in Arabidopsis and other Rosaceae, including *P. persica* and *R. chinensis*. As previously reported^21^, a clear distinction was obtained between the AP2-type and TOE-type clades of euAP2, including also the three *P. mume* euAP2 sequences found on chr1: PmuVar_Ch1_1236 clustered with Arabidopsis AP2 and TOE3, two members of the AP2 subfamily of euAP2; PmuVar_Ch1_1333 resulted orthologue of PET genes^13^ and will be hereafter referred to as pmPET, while PmTOE belongs to a different TOE-like clade closer to Arabidopsis TOE1 and TOE2, comprising Prupe.6G091100 (Fig. 2). Protein sequence comparison of the three TOE-type genes from mei and peach (Supplementary File 1C) suggests that pmPET and the PETALOSA Prupe.6G242400, PmTOE and the TOE-like Prupe.6G091100 all contain the functional euAP2 domains: two Ethylene-responsive element binding factor-associated amphiphilic repression motif (EAR-motif), a nuclear localization signal (NLS), two AP2 DNA binding domains (AP2-R1 and AP2-R2) with a linker region. TOE-like peptides from the remaining clade, PmuVar_Ch5_2600 and Prupe.2G220100 instead appear to lack a functional N-ter EAR motif and the AP2-R2 domain.

### Search of ***pmTOE***^***DEL***^ in resequenced accessions and identification of two ***pmPET*** candidate variants.

**Figure 3:**
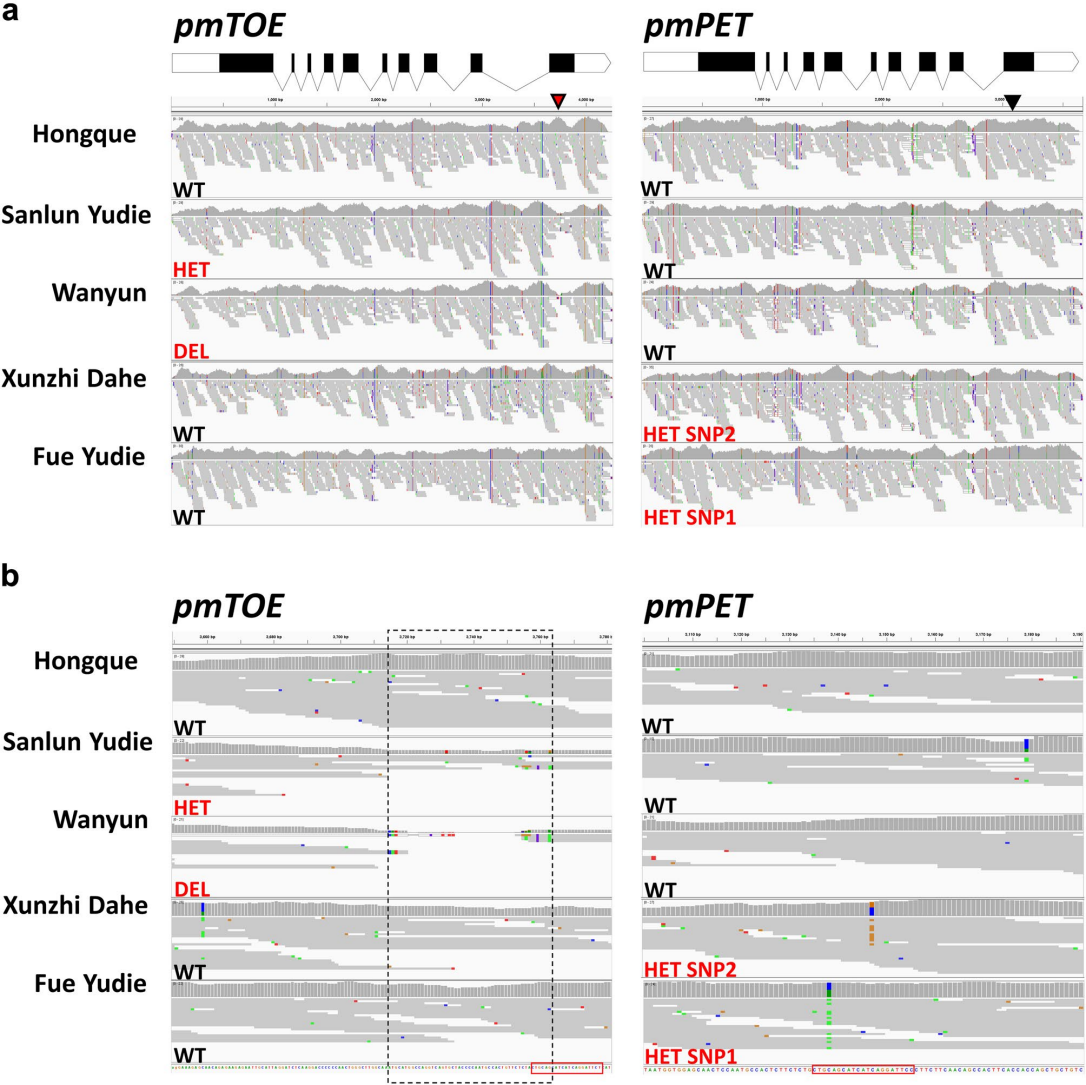
SRA reads coverage of *pmTOE* and *pmPET* in resequenced *P. mume* accessions. (**a**) Schematic representation of *pmTOE* and *pmPET* gene structures with coding exons as black boxes and reads coverage of five representative re-sequenced accessions Hongque (SRR5052904), Sanlun Yudie (SRR5046633), Wanyun (SRR5046692), Xunzhi Dahe (SRR5046661), Fue Yudie (SRR5052868). The corresponding genotype for *pmTOE* (WT) or *pmTOE*^*DEL*^ (DEL) at the position indicated with a red arrowhead, or for an heterozygous (HET) SNP in *pmPET* at the position indicated with a black arrowhead is reported at the bottom left corner of each stack. (**b**) Closer inspection of the on the regions indicated with the arrowheads in (**a**), miR172 target sites on both genes are highlighted with a red box. The dotted box delimits the 49 bp deletion in *pmTOE*^*DEL*^.

To confirm the presence of the *pmTOE*^*DEL*^ variant in the mei germplasm a Blast-based search was performed using whole-genome data from SRA^6^ along with available flower phenotype^3,20^. After discarding low-coverage libraries, a total of 209 mei re-sequenced accessions samples were retrieved: 53 out of 56 accessions belonging to the ‘Single Flowered’ group resulted homozygous for the *pmTOE* allele (Supplementary Table 1) while the *pmTOE*^*DEL*^ allele, either homozygous or heterozygous (Fig. 3a), was found in 135 out of 153 DF accessions from various groups reported as DF (‘Double Flowered’, Albo-plena, Cinnabar Purple, Green calyx, Meiren, Pendulous, Versicolor and Tortuosa). The discrepancies observed in 17 out of 153 accessions belonging to DF groups suggested the presence of other variants affecting the miR172/TOE-type genes module in addition to *pmTOE*^*DEL*^. This hypothesis was supported by the presence of GWAS signals associated to petal number on the unanchored scaffolds and in the middle of chromosome 1 in the same dataset^6^. Interestingly, two SNP variants were identified within the miR172 binding site of a *pmPET* ortholog located on the same chromosome (*PmuVar_Ch1_1333*, Fig. 1): C to A (named *pmPET*^*SNP1*^) and C to G (named *pmPET*^*SNP2*^) (Fig. 3b) in 16 out of 18 DF cultivars (Supplementary Table 1).

**Figure 4:**
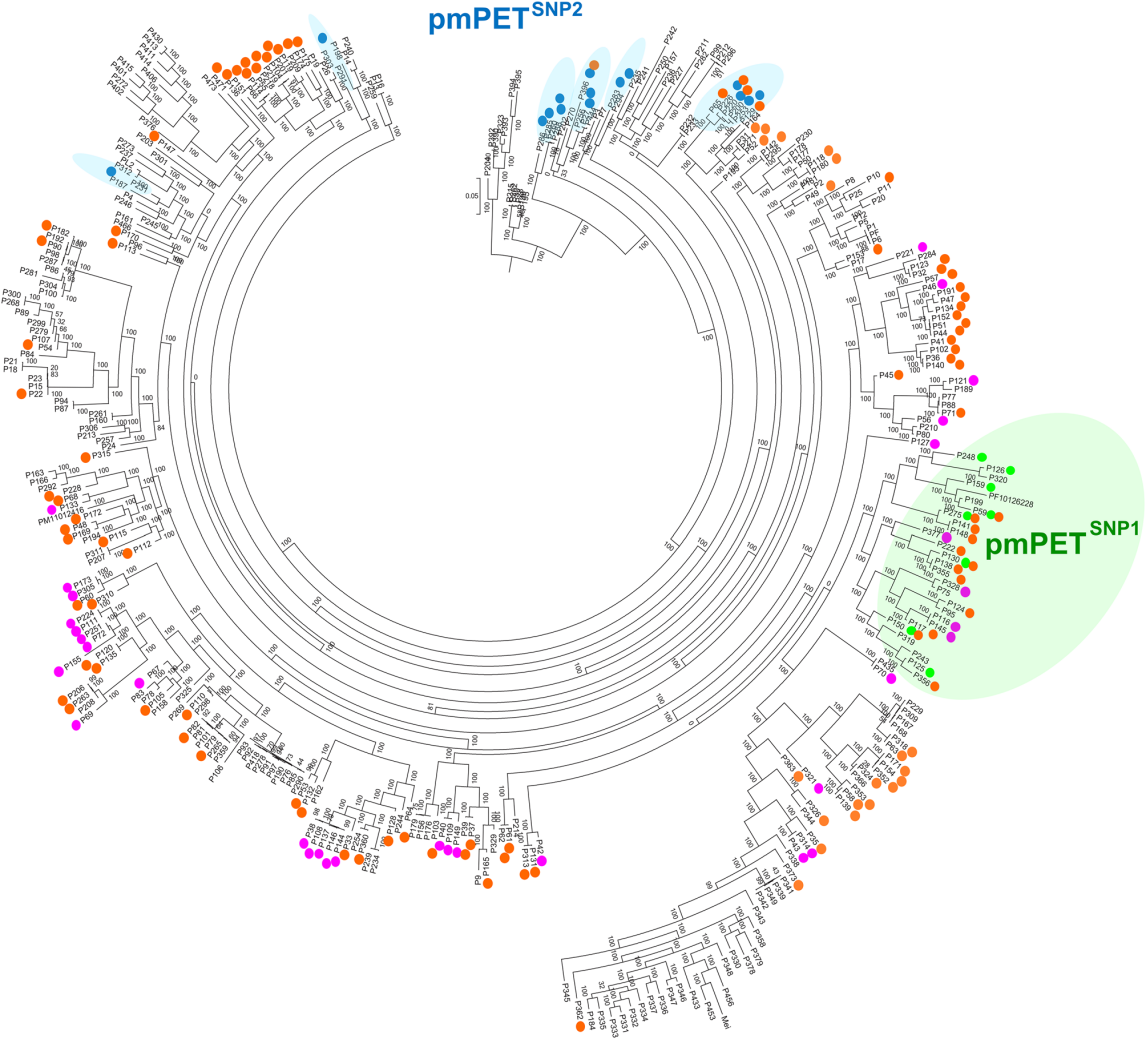
Distribution of *pmTOE*^*DEL*^, *pmPET*^*SNP1*^ and *pmPET*^*SNP2*^ alleles in the mei germplasm. Genotyped individuals scored as *pmTOE*^*DEL*^*/ pmTOE*^*DEL*^ (purple dots), *pmTOE/ pmTOE*^*DEL*^ (orange dots), *pmPET/ pmPET*^*SNP1*^ or *pmPET*^*SNP1*^/ *pmPET*^*SNP1*^ (green dots) and *pmPET/ pmPET*^*SNP2*^ (blue dots) were highlighted in a phylogenetic tree of 351 *P. mume* individuals obtained using genome-wide SNPs (modified from Ref.^6^).

The consistency of these mutations was then evaluated by graphically annotating the spread of the various alleles within the phylogenetic tree of a large mei collection of over 351 re-sequenced accessions^6^. Observing the clustering of the *pmTOE*^*DEL*^, *pmPET*^*SNP1*^ and *pmPET*^*SNP2*^ alleles and the positioning of genotyped samples (Fig. 4) strongly supports the hypothesis of sampling or assignment errors. For example, the presence of *pmTOE*^*DEL*^ in 3 SF accessions (#182, #251 and #352) contrasts with the strong genetic similarity between #251 and DF #72 (both carrying on the same *pmTOE*^*DEL*^*/pmTOE*^*DEL*^ alleles) or #352 with DF accessions #154 and #171 (heterozygous for *pmTOE/pmTOE*^*DEL*^). Instead, for the remaining two DF accessions recorded as ‘Pink Double’ but bearing no *pmTOE*^*DEL*^ or *pmPET*^*SNP*^ alleles (#50 and #379), the presence of a yet unidentified mutation responsible for the DF phenotype could not be excluded.

### *pmTOE* alleles and *pmPET* are transcribed

**Figure 5:**
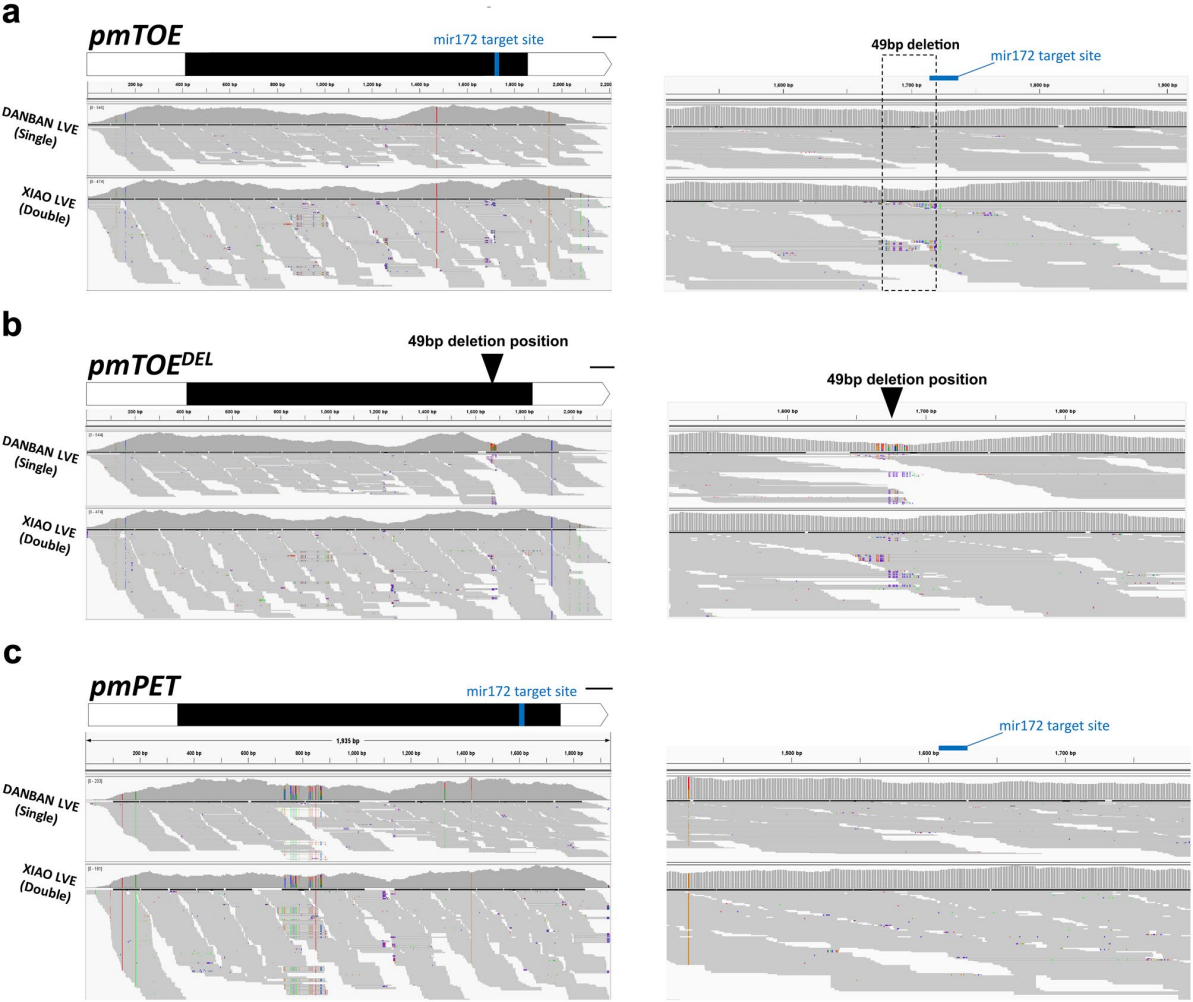
RNA-seq SRA reads coverage of *pmTOE*, *pmTOE*^*DEL*^ and *pmPET* predicted transcripts. Schematic representation at two different magnification of *pmTOE* (**a**), *pmTOE*^*DEL*^ (**b**) and *pmPET* (**c**) predicted transcript structures (CDS in black, miR172 target sequence in blue, position of the 49 bp deletion as an arrowhead, bar = 100 bp) and read coverage of RNA-seq data^22^ obtained from bud tissues of SF mei vaiety ‘Danban LVE’ (SRR19608366) and DF mei variety ‘Xiao Lve’ (SRR19608359).

To asses if *pmPET* and both *pmTOE* alleles are expressed at the mRNA level, RNA seq data from flower buds (SRR19608366 and SRR19608359, Ref.^22^) of single flowered ‘Danban Lve' and double flowered ‘Xiao Lve' (heterozygous for *pmTOE*^*DEL*^, sample #165) were mapped against the predicted transcripts of reference alleles. The reads coverage clearly shows that only *pmTOE* expression is detectable in the single accession ‘Danban Lve' while in DF ‘Xiao Lve' both *pmTOE* and *pmTOE*^*DEL*^ mapped correctly at the mRNA level (Fig. 5) and are therefore both expressed. Mapping of the RNA seq reads of both genotypes on the *pmPET* gene indicates that this gene is also expressed in bud tissues (Fig. 5).

### Wet validation of the presence of the ***PmTOE***^***DEL***^ allele in commercial mei cultivars

**Figure 6:**
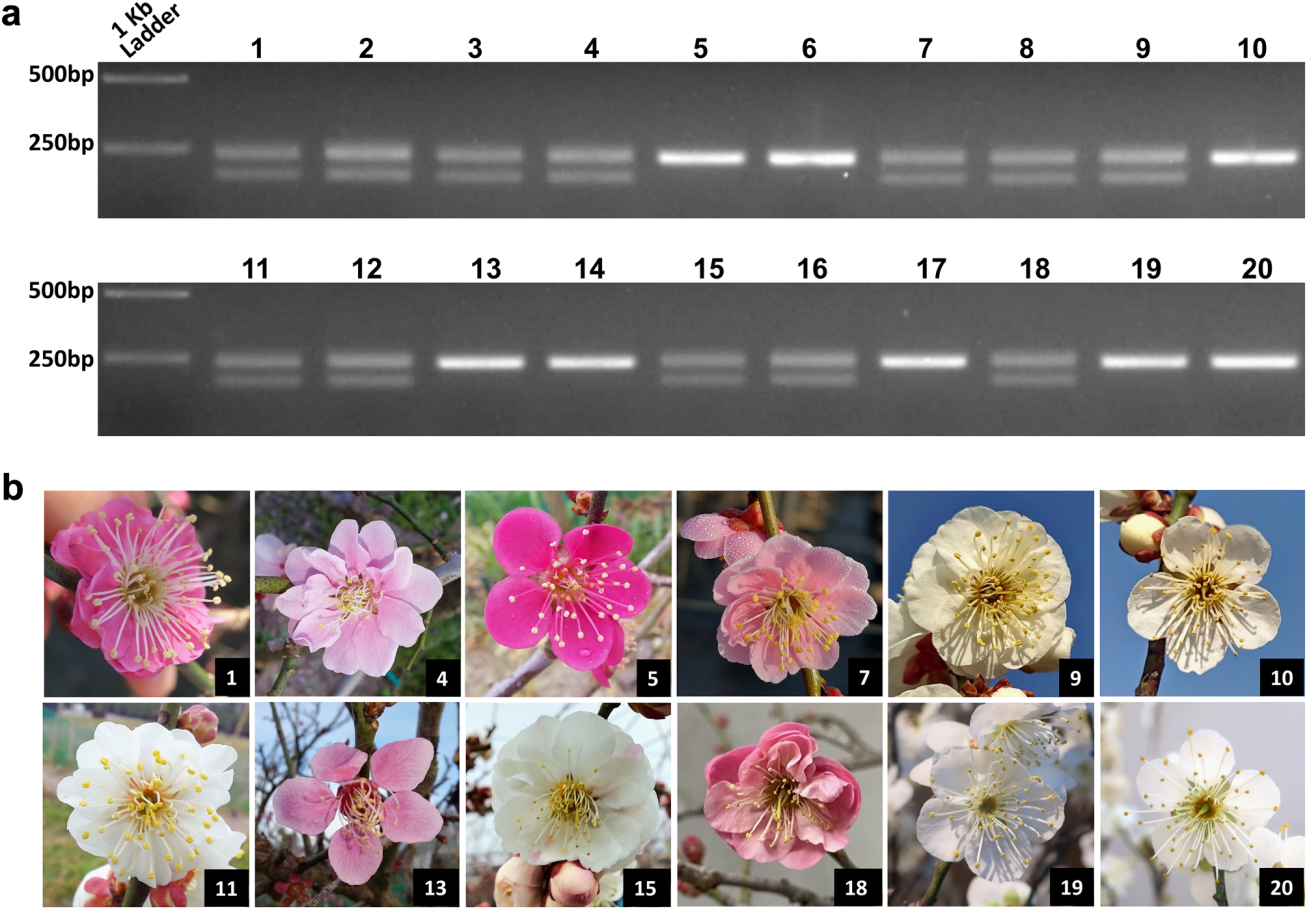
Molecular analysis of the *pmTOE* allele in different varieties. (**a**) PCR products obtained with the primers *pmTOE_Mk* using genomic DNA of DF mei varieties ‘Aliphandii’ (1, 2, 3), ‘Rosea Plena’ (4), ‘Pendula’ (7,8), ‘Bianco doppio’ (9), ‘Alboplena’ (11, 12), ‘Bicolor’ (15, 16), ‘Bonsai double RM’ (18) and SF varieties ‘Beni-chidori’ (5, 6), ‘Bianco Singolo’ (10), ‘Dwarf single’ (13, 14), ‘Bonsai single RM’ (17), ‘Bonsai single MI-1’ and ‘MI-2’ (19, 20), see Supplementary Table 2 for details. (**b**) Flower phenotype of representative varieties genotyped.

To validate the presence of the 49 bp indel variant within *PmTOE*^*DEL*^ gene candidate, a PCR assay was developed by designing specific primers (pmTOE_Mk). A total of 20 accessions were collected, of which 13 DF and 7 SF. As shown in Fig. 6, *PmTOE*^*DEL*^ allele was heterozygous in 13 DF accession while all the SF varieties only carried the *PmTOE* alleles. Sanger sequencing was further performed to confirm the identity of both alleles at miR172 recognition site of *pmTOE* and verify that all samples carried only wt *pmPET* alleles.

## Discussions

The mechanisms behind the dominant DF trait are of great interest for both plant researchers and breeders, in view of the economic value of the ornamental flower market. Working models based on key mutations were described also for members of the rose family^11–13^. With the availability of genomic resources and information from recent mapping studies, and based on the knowledge gathered in related species, it was possible to put the spotlight on a strong candidate *P. mume* euAP2 gene and a functional variant in one of its alleles as the main determinant of the trait in mei. The DF phenotype was previously found to be linked to loss of regulation of members of the PET clade of TOE euAP2 transcription factors^11–14^, and it was suggested that this was due to a prolonged activity in the floral meristem where they would normally act as upstream regulators of AGAMOUS (AG, Ref.^23^) and restrict its expression to the inner floral whorls. AG-like factors also act in shaping meristem size by negatively regulating homeodomain transcription factor WUSCHEL (WUS)^17,24^, and a mutation in the AG homolog in *P. lannesiana* was found responsible for flower doubleness in this species^25^. In peach the loss of regulation of the euAP2 function cascading in larger flowers with more petals can be brought about by either the disruption of the gene encoding *miR172d*, regulating euAP2 activity in the meristem, or mutations at the miR172 recognition site of PET gene *Prupe.6G242400*, impairing the ability of its mRNA to be silenced in this way. In the second scenario the mutation is dominant, as persistence of the transcript of one of the two alleles is sufficient to confer the phenotype. Independent mapping works^6,8,10^ provided the position and markers tightly associated to the DF trait in the mei germplasm on chromosome 1, ruling out the possibility that mutations on the gene orthologous to that encoding miR172d in peach were responsible in those materials, as this is found on chromosome 5 of the tortuosa genome (5: 25,786,770). Also considering the reported dominant inheritance of the trait^10^ euAP2 genes on chromosome 1 were therefore prime candidates for closer inspection, and one of them, *pmTOE*, fell within the genomic region under scrutiny. AP2-type *PmuVar_Ch1_1236* is also on chromosome 1, but studies in Arabidopsis indicate that expression of miR172-resistant versions of either AP2 or TOE3 (AP2-type euAP2 transcription factors) under their native promoters resulted in enlarged floral meristems and the production of supernumerary stamens, whereas supernumerary petals and an indeterminate floral meristem were obtained only when a constitutive promoter was used^17,26,27^, suggesting they have no direct role in determining floral patterning, but their miss-expression could perturb the activity of TOE-type euAP2 genes during flower development. This was substantiated in a recent study^28^ which also confirmed previous reports that miR172-mediated regulation of these genes occurs through the modulation of protein translation rather than post-transcriptional mRNA degradation^26,29,30^. As compared to the counterpart from a wild *P. mume* genome and the sequences from closely related species *P. persica* and *P. armenica* (Supplementary File 1), the *pmTOE* sequence from the genome of DF cultivar *tortuosa*, *pmTOE*^*DEL*^, revealed a 49 bp deletion affecting the miR172d target site, similar to those found to be sufficient to give the phenotype when found in PET genes from peach, roses and dianthus, or induced in tobacco^11–14^. *pmTOE* is not, however, the orthologue of PET genes in other species but of *Prupe.6G091100*, one of two other closely related TOE-type genes found in the mei and peach genome. A recent manuscript published when the present work was under review identified the same 49 bp deletion variant in *pmTOE* (named *PmAP2L* in that work) as responsible for the double-flower phenotype in mei, also providing functional analyses to demonstrate its pivotal role in controlling flower morphology^31^. By analysing publically available data we confirmed a predominance of the *pmTOE*^*DEL*^ variant, found in 135 out of 153 accessions from DF cultivar groups. Furthermore, single base substitutions at the miR172 recognition site of *pmPET* (also located on chromosome 1) were detected in a subset of DF accessions, helping to explain flower doubleness in 16 out of 18 DF *pmTOE/pmTOE* genotypes. The effects of single nucleotide mutations altering the miR172 binding site of PETALOSA gene orthologs have already been shown to induce extra-numerary petals in gene-edited tobacco plants^13^ and it is therefore plausible that *pmPET*^*SNP1*^ and *pmPET*^*SNP2*^ represent alternative alleles leading to flower doubleness in the mei germplasm, alongside *pmTOE*^*DEL*^ that we found more commonly associated with the trait. These kind of mutations are known to work at the mRNA level^26,28–30^, however as a result of translation, *pmTOE*^*DEL*^ and *pmPET*^*SNP1*^ would also result in a deletion and/or frame-shift of the N-ter end of the translated peptides, while *pmPET*^*SNP2*^ is predicted to result in a missense mutation directly causing a truncated protein product, although all functional euAP2 domains are predicted to be retained (Supplementary File 1C). *pmTOE*^*DEL*^ allele, either homozygous or heterozygous is well distributed across the various phylogenetic clusters, while *pmPET*^*SNP2*^ allele is shared by only five subgroups (including individuals sampled in France, Japan and different regions of China). Instead, *pmPET*^*SNP1*^ allele was found in a single cluster only comprising Chinese accessions, suggesting it may have originated more recently. The few cases of variant-trait inconsistency in available datasets are likely due to sampling, labeling or assignment errors.

*pmTOE* and *pmPET*, like their hortologues in peach (*Prupe.6G091100* and *Prupe.6G242400* respectively) are predicted to encode euAP2 peptides carrying all the functional domains, unlike the third TOE-type gene in both species *PmuVar_Ch5_2600* and *Prupe.2G220100*. In *P. persica* it was previously reported that both *Prupe.6G091100* and *Prupe.6G242400* were expressed at similar levels in flower buds, while no evidence of the presence of the transcript of *Prupe.2G220100* was found^11^. There is, to date, no evidence that mutations in *Prupe.6G091100* cause DF in peach but the present paper on a related species raises the possibility that the products of two genes have redundant function or they could affect different networks in an overlapping spatio-temporal frame, and the effects of escaping miR172 regulation by either gene would have similar effects on flower shape. In tetraploid petunia, simultaneous KO of both PET genes *BEN* and *BOB* was sufficient to induce a strong reduction of second whorl petal development^32^, suggesting that the PET function is not redundant with that of other TOE-type genes of this species, but in the same paper the authors concluded that the molecular mechanisms of floral A-function genes controlling perianth identity are not well-conserved between Asterids and a Rosids and great care should be taken when comparing models built in phylogenetically distant plants. It is therefore possible that mutations at the miR172 binding site of *Prupe.6G091100* could also lead to double flowers in peach, but such mutations have not yet been found. In case of the two ‘Pink Double’ accessions not bearing either *pmTOE*^*DEL*^, *pmPET*^*SNP1*^ or *pmPET*^*SNP2*^ , the presence of a yet unidentified DF-causing mutation could not be excluded, since the full spectrum of possible mutations might not have been unveiled. In other Rosaceae, some larger structural variants affecting the PET genes were underlining the DF phenotype: in peach, a 994 bp pet deletion depriving the last exon harboring the miR172 target site and in rosa a transposon insertion resulting in the loss of coding sequence encoded by the near entirety of the last two coding exons of the gene^11^ :such larger variants, whether present in some mei accessions would not be easily detectable in short reads sequencing libraries.

## Conclusions

Strong of the knowledge of genetic determinism pf the DF phenotype in other species and on the genomic location of causative variant in mei, we identified mutations in TOE-type euAP2 genes strongly linked to the trait. While SNP variants in the miR172 binding site of *pmPET* were found, the most widespread candidate variant was identified as a 49 bp deletion in *pmTOE*. This discovery suggests that *pmTOE-like* sequences should be included as candidate genes for DF in other species and entails further studies on the functions and interactions between euAP2 genes.

## Methods

### Sample collection and PCR analysis

Leaf samples of the 20 mei accessions used in this study were kindly provided by private nurseries and are listed in Supplementary Table 2. 16 samples were obtained from standard commercial trees while 4 samples were obtained from trees kept as bonsai. Accessions used are referred to with local denominations and can be divided into either SF or DF. Genomic DNA was extracted from 200 μg of young leaf tissue using the DNeasy Plant Kit (Qiagen) and 20 ng for each sample were used in PCR reactions using GoTaq PCR Master Mix (Promega) in a total volume of 10 ul and the appropriate primer combinations: *pmTOE_Mk_F* 5'-GAAAGAGCAACAGAGAAGAG-3' and *pmTOE_Mk_R* 5'-TTGAGAAGTATTGGCGGCAG-3' to screen for the 49 bp variant in *pmTOE*, and *pmPET_seq_F* 5'-AAGAAGCATTGCAGGCTTCC-3' and *pmPET_seq_R* 5'-GATCCTCAAGTTTCATAAGC-3' to amplify and sequence the miR172 target region in *pmPET*.

### Sequence-based genotyping

For sequencing-based method, NCBI SRA databases were Blast-searched with the reference alleles of interest, and for a deeper inspection resequencing data were retrieved and FASTQ files were mapped with Bowtie2 against the reference sequences on the Galaxy Platform^33^ and visualized using IGV^34^. The reads were aligned against the genomic sequences of *pmTOE* and *pmTOE*^*DEL*^ to identify homozygous or heterozygous presence of the 49 bp deletion. The reads were also aligned against the sequence of *pmPET* to identify candidate variants in this gene. SRA data SRR5046711, SRR5046712, SRR5046754 and SRR5046758 were excluded from the analysis due to low coverage.

### Molecular phylogenetic analysis by maximum likelihood method

Phylogenetic relations of *P. mume* euAP2 peptide sequences and orthologous sequences from *P. persica*, *R. chinensis* and *A. thaliana* from previously published work^11^ were estimated in MEGAX^35^. Peptide sequences were aligned by MUSCLE with default settings. Evolutionary relationships among euAP2 peptides were inferred by using the Maximum Likelihood method based on the JTT matrix-based model. The rate variation model allowed for some sites to be evolutionarily invariable and a discrete Gamma distribution was used to model evolutionary rate differences among sites. The reliability of the phylogenetic tree was estimated by setting 200 bootstrap replicates.

### Declaration on ethics and research permits

All procedures were conducted in accordance with the guidelines and legislation. No permissions or licenses were required. *P. mume* leaf samples used in this study were either from commercial varieties kindly provided by nurseries or from local bonsai growers and the details are found in the manuscript. DNA samples are available on reasonable request from the corresponding author.

### Supplementary Information

Supplementary Information.

Supplementary Tables.

## Data Availability

The datasets analyzed in the current study were already available on NCBI as Bioprojects PRJNA352648^6^ and PRJNA847636^22^.
