# Supplementary material for: Mutations overlying the miR172 target site of TOE-type genes are prime candidate variants for the double-flower trait in mei
Source: Sci Rep. 2024 Mar 27;14:7300. doi: 10.1038/s41598-024-57589-8 (PMC10973477; doi:10.1038/s41598-024-57589-8)

Mutations overlying the miR172 target site of TOE-type genes are prime candidate variants for the double-flower trait in mei

Stefano Gattolin, Elisa Calastri, Maria Rosaria Tassone, Marco Cirilli

Supplementary Information

Supplementary Information File 1

Sequence information of the *pmTOE* and *pmPEt al*leles identified in this work. (A) Alignment of genomic sequences of mei *pmTOE*, *pmTOE*^{DEL} and the orthologue sequences from peach and apricot. Predicted start and stop codons are highlighted in black. miR172 recognition site is boxed, sequence corresponding to the 49 bp deletion in *pmTOE*^{DEL} was highlighted in grey in the other sequences. (B) Genomic, cDNA and predicted protein sequences of *pmTOE*^{DEL} and *pmTOE* and sequence comparison of predicted protein sequences. Exons are in bold, UTRs and introns in grey. (C) Protein sequence comparison of the three TOE-type genes from mei and peach. The functional euAP2 domains are highlighted in yellow (EAR motifs), green (nuclear localization signals, NLS), light blue (AP2 DNA binding domains, AP2-R1 and AP2-R2) and grey (linker region). (D) Genomic sequence of *pmPET* and alignment of the sequences of *pmPET*, *pmPET*^{SNP1} and *pmPET*^{SNP2} surrounding the miR172 target site (boxed), with the effect on the predicted peptide sequences. SNPs are highlighted in grey. (E) Uncropped gel image of PCR analysis (Fig 6).

Supplementary Dataset file 1.

Supplementary Table 1: Blast-based genotyping of the *pmTOE*^{DEL}, *pmPET*^{SNP1} and *pmPET*^{SNP2} alleles. Genotypes were obtained using whole-genome data from SRA [6] along with available flower phenotype of 209 mei re-sequenced accessions. **Supplementary Table 2 :** Genotyping of the *PmTOE* allele and *pmPET* alleles in commercial mei cultivars. A total of 20 accessions collected in Italy of which 13 DF and 7 SF were assayed for presence of *PmTOE* and/or *PmTOE*^{DEL} by PCR assay using *pmTOE_Mk*, and for presence of *pmPET*^{SNP1} and/or *pmPET*^{SNP2} by Sanger sequencing.

Supplementary Information file 1

A

```
Apricot_PaJTYG0600022743     GGTCAGCTGACTGAGCTGAAAAGTCTAGAACCGGAGCTGAATTTAGAGGG
Mei_pmTOE                    GGTCAGCTGACTGAGCTGAAAAGTCTAGAACCGGAGCTGAATTTAGAGGG
Mei_pmTOE^DEL                GGTCAGCTGACTGAGCTGAAAAGTCTAGAACCGGAGCTGAATTTAGAGGG
Peach_Prupe.6G091100         GGTCAGCTGACTGAGCTGAAAAGTCTAGAACCGGAGCTGAATTTAGAGGG
                             **************************************************

Apricot_PaJTYG0600022743     ACTAAAATTTATATTAAGAGAGCTGAGCTATGCTGGATCTTAATCTGAAC
Mei_pmTOE                    ACTAAAATTTATATTAAGAGAGCTGAGCTATGCTGGATCTTAATCTGAAC
Mei_pmTOE^DEL                ACTAAAATTTATATTAAGAGAGCTGAGCTATGCTGGATCTTAATCTGAAC
Peach_Prupe.6G091100         ACTAAAATTTATATTAAGAGAGCTGAGCTATGCTGGATCTTAATCTGAAC
                             **************************************************

Apricot_PaJTYG0600022743     GTCGTCGGTTCTGGCCCAAACGACGTCGAGTCGTGTGGCACCCAAATGGA
Mei_pmTOE                    GTCGTCGGTTCTGACCCAAACGACGTCGAGTCATGTGGCACCCAAATGGA
Mei_pmTOE^DEL                GTCGTCGGTTCTGACCCAAACGACGTCGAGTCATGTGGCACCCAAATGGA
Peach_Prupe.6G091100         GTCGTCGGTTCTGGCCCAAACGACGTCGAGTCGTGCGGCACCCAAATGGA
                             *************.******************.** **************

Apricot_PaJTYG0600022743     CGAGTCGGGGACGTCGAACTCGTCCGTGGTCAATGCCGACGCATCCAGCA
Mei_pmTOE                    CGAGTCGGGGACGTCGAACTCGTCCGTGGTCAATGCCGACGCATCCAGCA
Mei_pmTOE^DEL                CGAGTCGGGGACGTCGAACTCGTCCGTGGTCAATGCCGACGCATCCAGCA
Peach_Prupe.6G091100         CGAGTCGGGGACGTCGAACTCGTCCGTGGTCAATGCCGACGCATCCAGCA
                             **************************************************

Apricot_PaJTYG0600022743     CCAACGACGACTCGTGCTCCACACGCGCCGCCAGATACGACGCCGTCACG
Mei_pmTOE                    CCAACGACGACTCGTGCTCCACACGCGCCGCCAGATACGACGCCGTCACG
Mei_pmTOE^DEL                CCAACGACGACTCGTGCTCCACACGCGCCGCCAGATACGACGCCGTCACG
Peach_Prupe.6G091100         CCAACGACGACTCGTGCTCCACACGCGCCGCCAGATACGACGCCGTCACG
                             **************************************************

Apricot_PaJTYG0600022743     ACCTTCAACTTCGATATTCTCAAGGTCAGGGGCGGAGAAGATGAAGAAGA
Mei_pmTOE                    ACCTTCAACTTCGATATTCTCAAGGTCAGGGGCGGAGAAGATGAAGAAGA
Mei_pmTOE^DEL                ACCTTCAACTTCGATATTCTCAAGGTCAGGGGCGGAGAAGATGAAGAAGA
Peach_Prupe.6G091100         ACCTTCAACTTCGATATTCTCAAGGTCAGGGGCGGAGAAGATGAAGAAGA
                             **************************************************

Apricot_PaJTYG0600022743     CGATGTCGTCGTGACTAAGGAGCTGTTCCCGGTCACCGGGGGCCTGAGCA
Mei_pmTOE                    CGATGTCGTCGTGACTAAGGAGCTGTTCCCGGTCACCGGGGCCCTGAGCA
Mei_pmTOE^DEL                CGATGTCGTCGTGACTAAGGAGCTGTTCCCGGTCACCGGGGCCCTGAGCA
Peach_Prupe.6G091100         CGATGTCGTCGTGACTAAGGAGCTGTTCCCGGTCACCGGGGGCCTGAGCA
                             ***************************************** ********

Apricot_PaJTYG0600022743     ATTGGCCCGGCCAGGGGCAGTCGTCAGCGTCGTCGTCTTTGGTGAGGAAG
Mei_pmTOE                    ATTGGCCCGGCCAGGGGCAGTCGTCAGCGTCGTCGTCTTTGGTGAGGAAG
Mei_pmTOE^DEL                ATTGGCCCGGCCAGGGGCAGTCGTCAGCGTCGTCGTCTTTGGTGAGGAAG
Peach_Prupe.6G091100         ATTGGCCCGGCCAGGGGCAGTCGTCAGCGTCGTCGTCTTTGGTGAGGAAG
                             **************************************************

Apricot_PaJTYG0600022743     AACTTGATGGAGCTTGGGTTCGATCATGGCGGGTCCGGAGAGGTCAGGTT
Mei_pmTOE                    AACTTGATGGAGCTTGGGTTCGATCATGGCGGGTCCGGAGAGGTCAGGTT
Mei_pmTOE^DEL                AACTTGATGGAGCTTGGGTTCGATCATGGCGGGTCCGGAGAGGTCAGGTT
Peach_Prupe.6G091100         AACTTGATGGAGCTTGGGTTCGATCATGGCGGGACCGGAGAGGTCAGGTT
                             *********************************:****************

Apricot_PaJTYG0600022743     GGTTCAACAGAAACAACAGCAACCAGCCGCACCGCCACCACAGCAACAGC
Mei_pmTOE                    GGTTCAACAGAAACAACAGCAACCAGCCGCACCGCCACCACAGCAACAGC
Mei_pmTOE^DEL                GGTTCAACAGAAACAACAGCAACCAGCCGCACCGCCACCACAGCAACAGC
Peach_Prupe.6G091100         GGTTCAACAGAAACAACAGCAACCAGCCGCACCGCCACCACAGCAACAGC
                             **************************************************

Apricot_PaJTYG0600022743     AGGTGAAGAAGAGCAGAAGAGGGCCGAGGTCTCGGAGCTCTCAGTATAGA
Mei_pmTOE                    AGGTGAAGAAGAGCAGAAGAGGGCCGAGGTCTCGGAGCTCTCAGTATAGA
Mei_pmTOE^DEL                AGGTGAAGAAGAGCAGAAGAGGGCCGAGGTCTCGGAGCTCTCAGTATAGA
Peach_Prupe.6G091100         AGGTGAAGAAGAGCAGAAGAGGGCCGAGGTCTCGGAGCTCTCAGTATAGA
                             **************************************************

Apricot_PaJTYG0600022743     GGGGTCACCTTCTATAGAAGAACTGGTAGATGGGAATCTCATATTTGGTT
Mei_pmTOE                    GGGGTCACCTTCTATAGAAGAACTGGTAGATGGGAATCTCATATTTGGTT
Mei_pmTOE^DEL                GGGGTCACCTTCTATAGAAGAACTGGTAGATGGGAATCTCATATTTGGTT
Peach_Prupe.6G091100         GGGGTCACCTTCTATAGAAGAACTGGTAGATGGGAATCTCATATTTGGTT
```

```
                              **************************************************

Apricot_PaJTYG0600022743      AGTCCAATTTTGATTTCCTTAATATGGTTCTATTGGGTTTCATGTTTCGT
Mei_pmTOE                     AGTCCAATTTTGATTTCCTTAATATGGTTCTATTGGGTTTCATGTTTCGT
Mei_pmTOE^DEL                 AGTCCAATTTTGATTTCCTTAATATGGTTCTATTGGGTTTAATGTTTCGT
Peach_Prupe.6G091100          AGTCCAATTTTGATTTCCTTAATATGGTTCTATTGGGTTTCATGTTTCGT
                              ****************************************.*********

Apricot_PaJTYG0600022743      TACTTGGATTTGCTTATTTTAATGTTG--------------TTTCTTTTG
Mei_pmTOE                     TACTTGGATTTGCTTATTTTAATGTTG--------------TTTCTTTTG
Mei_pmTOE^DEL                 TACTTGGATTTGCTTATTTTAATGTTG--------------TTTCTTTTG
Peach_Prupe.6G091100          TACTTGGATTTGCTTATTTTATTCTAATTTTTTAGGGTTGTTTTCTTTTG
                              *********************:* *:.              *********

Apricot_PaJTYG0600022743      GGTTGAGTTTGAATTGCAATTATGCAAATGCATCTGTTTCATATAGTATT
Mei_pmTOE                     GGTTGAGTTTGAATTGCAATTATGCAAATGCATCTGTTTCATATAGTATT
Mei_pmTOE^DEL                 GGTTGAGTTTGAATTGCAATTATGCAAATGCATCTGTTTCATATAGTATT
Peach_Prupe.6G091100          TGTTGAGTTTGAATTGCAATTATGCAAATGCATCTGTTTCATATAGTATT
                               *************************************************

Apricot_PaJTYG0600022743      AATCCGATTTACTAATTATTTTTAATTTTTGCTGGGTGCAGGGATTGCGG
Mei_pmTOE                     AATCCGATTTACTAATTATTTTTAATTTTTGCTGGGTGCAGGGATTGCGG
Mei_pmTOE^DEL                 AATCCGATTTACTAATTATTTTTAATTTTTGCTGGGTGCAGGGATTGCGG
Peach_Prupe.6G091100          AATCCGATTTACTAATTATTTTTAATTTTTGCTGGGTGCAGGGATTGCGG
                              **************************************************

Apricot_PaJTYG0600022743      GAAACAAGTGTATTTGGGTATGTGTTTTTGACCTTTCCCATTTGATTAGC
Mei_pmTOE                     GAAACAAGTGTATTTGGGTATGTGTTTATGACCTTTCCAATTTGATTAGC
Mei_pmTOE^DEL                 GAAACAAGTGTATTTGGGTATGTGTTTATGACCTTTCCAATTTGATTAGC
Peach_Prupe.6G091100          GAAACAAGTGTATTTGGGTATGTGTTTTTGATCTTGCCCATTTGATTAGC
                              ***************************:*** *** **.***********

Apricot_PaJTYG0600022743      TTGGATTTAGTTATAGTAATTATTAAGGCAATTATTTGTCATGCTTTTCT
Mei_pmTOE                     TTGGATTTAGTTATAGTAATTATTGAGGCAATTATTTGTCATGCTTTTCT
Mei_pmTOE^DEL                 TTGGATTTAGTTATAGTAATTATTGAGGCAATTATTTGTCATGCTTTTCT
Peach_Prupe.6G091100          TTGGATTTAGTTATAGTAATTATTAAGGCAATTATTTGTCATGCTTTTCT
                              ************************.*************************

Apricot_PaJTYG0600022743      ATTTGGAAAGTTTTTGATGAAAATTTAAGTGTTGGGTTTGACATAGG-TG
Mei_pmTOE                     ATTTGGAAAGTTTTTTATGAAAATTTAAGTGTTGGGTTTGACATAGG-TG
Mei_pmTOE^DEL                 ATTTGGAAAGTTTTTGATGAAAATTTAAGTGTTGGGTTTGACATAGG-TG
Peach_Prupe.6G091100          ATCTGGAAAGCTTTTGATGAAAATTTAAGTGTTGGGTTTGACATTAGGTG
                              ** ******* **** ****************************:.* **

Apricot_PaJTYG0600022743      GATTTGACACTGCTCATGCTGCGGCTAGGTAAAAAGCCTTCTTCATATTT
Mei_pmTOE                     GATTTGACACTGCTCATGCTGCGGCTAGGTAAAAAGCCTTCTTCATATTT
Mei_pmTOE^DEL                 GATTTGACACTGCTCATGCTGCGGCTAGGTAAAAAGCCTTCTTCATATTT
Peach_Prupe.6G091100          GATTTGACACTGCTCATGCTGCGGCTAGGTAAAAAGCCATCTTCATATTT
                              **************************************:***********

Apricot_PaJTYG0600022743      TTTTGCTTGGATTTCGTACTGTTTGTGAATTTAGATAATTAGTCATTGTG
Mei_pmTOE                     TTTTGCTTGGATTTCGTACTGTTTGTGAATTTAGATAATTAGTCATTGTG
Mei_pmTOE^DEL                 TTTTGCTTGGATTTCGTACTGTTTGTGAATTTAGATAATTAGTCATTGTG
Peach_Prupe.6G091100          TTTTGCTTGGATTTCGTACTGTTTGTGAATTTAGATAATTAGTCATTCTG
                              *********************************************** **

Apricot_PaJTYG0600022743      GTTGCTACTGTATGTATTTTT-ATTAGTATTCTTAAATTGAGTTTGCTGA
Mei_pmTOE                     GTTACTACTGTATGTATTTTT-ATTAGTATTCTTAAATTGGGTTTGCTGA
Mei_pmTOE^DEL                 GTTACTACTGTATGTATTTTT-ATTAGTATTCTTAAATTGGGTTTGCTGA
Peach_Prupe.6G091100          GTTACTACTGTATGTATTTTTTATTAGTATTCTTAAATTGGGTTTGTTGA
                              ***.***************** ******************.***** ***

Apricot_PaJTYG0600022743      ATTTAGAGCCTACGATCGAGCTGCTATTAAGTTCAGAGGAGTTGATGCTG
Mei_pmTOE                     ATTTAGAGCCTACGATCGAGCTGCTATTAAGTTCAGAGGAGTTGATGCTG
Mei_pmTOE^DEL                 ATTTAGAGCCTACGATCGAGCTGCTATTAAGTTCAGAGGAGTTGATGCTG
Peach_Prupe.6G091100          ATTTAGAGCCTACGATCGAGCTGCTATTAAGTTTAGAGGAGTTGATGCTG
                              ********************************* ****************

Apricot_PaJTYG0600022743      ATATCAATTACAACCTCAGTGATTATGAGGAGGATTTGAAACAGGTAGGA
Mei_pmTOE                     ATATCAATTACAACCTCAGTGATTATGAGGAGGATTTGAAACAGGTAGGA
Mei_pmTOE^DEL                 ATATCAATTACAACCTCAGTGATTATGAGGAGGATTTGAAACAGGTAGGA
Peach_Prupe.6G091100          ATATCAATTACAACCTCAGTGATTATGAGGAGGATTTGAAACAGGTAGGA
                              **************************************************

Apricot_PaJTYG0600022743      TAATGCACTTGTTTCTTGATGAACCTATGTCTGCTTAATTTCATATGCTG
Mei_pmTOE                     TAATGCACTTGTTTCTTGATGAACCTATGTCTGCTTAATTTCATATGCTG
Mei_pmTOE^DEL                 TAATGCACTTGTTTCTTGATGAACCTATGTCTGCTTAATTTCATATGCTG
Peach_Prupe.6G091100          TAATGCACTTGTTTCTTGATGAACCTAAGTCTGCTTAATTTCATATGCTG
                              ***************************:**********************
```

```
Apricot_PaJTYG0600022743      AGTGGAATCATATTTTGATCTATTTGTAATGAATGAGCAGATGAAGAATT
Mei_pmTOE                     AGTGGAATCATATTTTGATCTATTTGTAATGAATGAGCAGATGAAGAATT
Mei_pmTOE^DEL                 AGTGGAATCATATTTTGATCTATTTGTAATGAATGAGCAGATGAAGAATT
Peach_Prupe.6G091100          AGTGGAATCATATTTTGATCTATTTTTAATGAATGAGCAGATGAAGAATT
                              ************************* ************************

Apricot_PaJTYG0600022743      TGACCAAGGAAGAATTTGTGCACATACTACGGAGGCAGAGCACTGGTTTC
Mei_pmTOE                     TGACCAAGGAAGAATTTGTGCACATACTACGGAGGCAGAGCACTGGTTTC
Mei_pmTOE^DEL                 TGACCAAGGAAGAATTTGTGCACATACTACGGAGGCAGAGCACTGGTTTC
Peach_Prupe.6G091100          TGACCAAGGAAGAATTTGTGCACATACTACGGAGGCAGAGCACTGGTTTC
                              **************************************************

Apricot_PaJTYG0600022743      TCGAGGGGGAGCTCGAGATATAGAGGGGTTACGCTGCACAAATGTGGCCG
Mei_pmTOE                     TCGAGGGGGAGCTCGAGATATAGAGGGGTTACGCTGCACAAATGTGGCCG
Mei_pmTOE^DEL                 TCGAGGGGGAGCTCGAGATATAGAGGGGTTACGCTGCACAAATGTGGCCG
Peach_Prupe.6G091100          TCGAGGGGGAGCTCGAGATATAGAGGGGTTACGCTGCACAAATGTGGCCG
                              **************************************************

Apricot_PaJTYG0600022743      ATGGGAAGCCCGAATGGGGCAGTTCCTTGGCAAAAAGTGAGGAATTATTT
Mei_pmTOE                     ATGGGAAGCTCGAATGGGGCAGTTCCTTGGCAAAAAGTGAGGAATTATTT
Mei_pmTOE^DEL                 ATGGGAAGCTCGAATGGGGCAGTTCCTTGGCAAAAAGTGAGGAATTATTT
Peach_Prupe.6G091100          ATGGGAAGCTCGAATGGGGCAGTTCCTTGGCAAAAAGTGAGGAATCATTT
                              ********* *********************************** ****

Apricot_PaJTYG0600022743      TATGGAATTTTGAATTTCTTACAGTAGGTTTCGGTTTAGACTTTATATGA
Mei_pmTOE                     TATGGAATTTTGAATTTCTTACAGTAGGTTTCGGTTTAGACTTTATATGA
Mei_pmTOE^DEL                 TATGGAATTTTGAATTTCTTACAGTAGGTTTCGGTTTAGACTTTATATGA
Peach_Prupe.6G091100          TATGGAATTTTGAATTTCTTACAGTAGGTTTCGGTTTAGACTTTATACGA
                              *********************************************** **

Apricot_PaJTYG0600022743      TCAGAGGTTGTCAATTTGAACTGAGAATTAATTTCGGATTAATTCTCAAT
Mei_pmTOE                     TCAGAGGTTGTCAATTTGAACTGAGAATTAATTTCGGATTAATTCTCAAT
Mei_pmTOE^DEL                 TCAGAGGTTGTCAATTTGAACTGAGAATTAATTTCGGATTAATTCTCAAT
Peach_Prupe.6G091100          TCAGAGGTTGTCGGTTTGAACTGAGAATTAATTTCGGATTAATTCTCAAT
                              ************..************************************

Apricot_PaJTYG0600022743      CAGTCGTTGTCTTCTCTTTTCCTTGTAAAAAAAAAAGAAAAAAAAAAGTC
Mei_pmTOE                     CAGTCGTTGTCTTCTCTTTTCCTTG---TAAAAAAAAAAAAAAAAAAGTC
Mei_pmTOE^DEL                 CAGTCGTTGTCTTCTCTTTTCCTTG-------TAAAAAAAAAAAAAAGTC
Peach_Prupe.6G091100          CAGTCGTTGTCTTCTCTTTTCCTTGTAAAAAAAAAAAAAAAAAAAAAGTC
                              *************************       :***.*************

Apricot_PaJTYG0600022743      CAAGTGCCTATTTCAAAGAAAAGAAATTGTGGGGACGGTCGTTTCATCAT
Mei_pmTOE                     GAAGTGCCTATTTCAAAGAAAAAAAAATTGTGGGGACGGTCGTTTCATCAT
Mei_pmTOE^DEL                 CAAGTGCCTATTTCAAAGAAAAAAAAATTGTGGGGACGGTCGTTTCATCAT
Peach_Prupe.6G091100          CAAGTGCCTATTTCAAAGAAAAAAAAATTGTGGGGACGGTCGTTTCATCAT
                               *********************.***************************

Apricot_PaJTYG0600022743      GGAGGTTACAATGCTTGCATGCAGGTATATATATCTTGGGCTATTCGACA
Mei_pmTOE                     GGAGGTTACAATGCTTGCATGCAGGTATATATATCTTGGGCTATTCGACA
Mei_pmTOE^DEL                 GGAGGTTACAATGCTTGCATGCAGGTATATATATCTTGGGCTATTCGACA
Peach_Prupe.6G091100          GGAGGTTACAATGCTTGCATGCAGGTATATATATCTTGGGCTATTCGACA
                              **************************************************

Apricot_PaJTYG0600022743      GTGAAGTAGAAGCTGCAAGGTCCTAATGTCATGAATTACACTCTACCTGA
Mei_pmTOE                     GTGAAGTAGAAGCTGCAAGGTCCTAATGTCATGAATTACACTCTACCTGA
Mei_pmTOE^DEL                 GTGAAGTAGAAGCTGCAAGGTCCTAATGTCATGAATTACACTCTACCTGA
Peach_Prupe.6G091100          GCGAAGTAGAAGCTGCAAGGTCCTAATGTCATGAATTACACTCTACCTGA
                              * ************************************************

Apricot_PaJTYG0600022743      CTGAATTTATTACCCTGAATTCTCAACCTCATTAGGCTTCCATCCAACAC
Mei_pmTOE                     CTGAATTTATTACCCTGAATTCTCAACCTCATTAGGCTTCCATCCAACAC
Mei_pmTOE^DEL                 CTGAATTTATTACCCTGAATTCTCAACCTCATTAGGCTTCCATCCAACAC
Peach_Prupe.6G091100          CTGAATTTATTACCCTGAATTCTCAACCTCATTAGGCTTCCATCCAACAC
                              **************************************************

Apricot_PaJTYG0600022743      ATCTCTCCCTTTTGATTTTTCTGGAAATTAGGGCTTATGACAAGGCAGCA
Mei_pmTOE                     ATCTCTCCCTTTTGATTTTTCTGGAAATTAGGGCTTATGACAAGGCAGCA
Mei_pmTOE^DEL                 ATCTCTCCCTTTTGATTTTTCTGGAAATTAGGGCTTATGACAAGGCAGCA
Peach_Prupe.6G091100          ATCTCTCCCTTTTGATTTTTCTGGAAATTAGGGCTTATGACAAGGCAGCA
                              **************************************************

Apricot_PaJTYG0600022743      ATCAAATGTAATGGAAGGGAAGCAGTCACCAACTTTGAGCCAAGCACATA
Mei_pmTOE                     ATCAAATGTAATGGAAGGGAAGCAGTCACCAACTTTGAGCCAAGCACATA
Mei_pmTOE^DEL                 ATCAAATGTAATGGAAGGGAAGCAGTCACCAACTTTGAGCCAAGCACATA
Peach_Prupe.6G091100          ATCAAATGTAATGGAAGGGAAGCAGTCACCAACTTTGAGCCAAGCACATA
                              **************************************************
```

```
Apricot_PaJTYG0600022743      TGAAGGGGAGATGATATCTGAGGCTGGTAATGAAGGTACGATTTCACAAA
Mei_pmTOE                     TGAAGGGGAGATGATATCTGAGGCTGGTAATGAAGGTACGATTTCACAAA
Mei_pmTOE^DEL                 TGAAGGGGAGATGATATCTGAGGCTGGTAATGAAGGTACGATTTCACAAA
Peach_Prupe.6G091100          TGAAGGGGAGATGATATCTGAGGCTGGTAATGAAGGTACGATTTTACAAA
                              ******************************************** *****

Apricot_PaJTYG0600022743      TATCACTATCTTTTGAGGTAGTTCAAATGCATCTCCTGTTTTCAAGGCAG
Mei_pmTOE                     TATCACTATCTTTTGAGGCAGTTCAAATGCATCTCCTGTTTTCAAGGCAG
Mei_pmTOE^DEL                 TATCACTATCTCTTGAGGTAGTTCAAATGCATCTCCTGTTTTCAAGGCAG
Peach_Prupe.6G091100          TATCACTATCTTTTGAGGTAGTTCAAACGCATCTCCTGTTTTCAAGGCAG
                              *********** ****** ******** **********************

Apricot_PaJTYG0600022743      GTGGTTTACTAATTAGAAAATTTTCAATAAATTTGTCTTCGTAGAATTGT
Mei_pmTOE                     GTGGTTTACTAATTAGAAAATTTTCAAGAAATTTGTCTTCGTAGAATTGT
Mei_pmTOE^DEL                 GTGGTTTACTAATTAGAAAATTTTCAAGAAATTTGTCTTCGTAGAATTGT
Peach_Prupe.6G091100          GTGGTTTACTAATTAGAAAATTTTCAAGAAATTTGTCTTCGTAGAATTGT
                              *************************** **********************

Apricot_PaJTYG0600022743      GTTTATATATATTTTTCTTCCTGATGCAGATGGCGATCACAATCTTGATC
Mei_pmTOE                     GTTTATATATTTTTTTCTCCCTGATGCAGATGGCGATCACAATCTTGATC
Mei_pmTOE^DEL                 GTTTATATATTTTTTTCTCCCTGATGCAGATGGCGATCACAATCTTGATC
Peach_Prupe.6G091100          GTTTATATATATTTTTCCCCCTGATGCAGATGGCGATCACAATCTCGATC
                              **********:******  ************************** ****

Apricot_PaJTYG0600022743      TGAATTTGGGGATATCTCCCCCTTCATTTGGCAATTGTCAAAAGGAAGTC
Mei_pmTOE                     TGAATTTGGGGATATCTCCCCCTTCATTTGGCAATTGTCAAAAGGAAGTC
Mei_pmTOE^DEL                 TGAATTTGGGGATATCTCCCCCTTCATTTGGCAATTGTCAAAAGGAAGTC
Peach_Prupe.6G091100          TGAATTTGGGGATATCTCCCCCTTCATTTGGCAATTGTCAAAAGGAAGTC
                              **************************************************

Apricot_PaJTYG0600022743      GAGGGGCATCTTCAATTCCATTCCGGCCCTTATGATGGGCACAATGGAAA
Mei_pmTOE                     GAGGGGCATCTTCAATTCCATTCCGGCCCTAATGATGGGCACAATGGAAA
Mei_pmTOE^DEL                 GAGGGGCATCTTCAATTCCATTCCGGCCCTTATGATGGGCACAATGGAAA
Peach_Prupe.6G091100          GAGGGGCATCTTCAATTCCATTCCGGCCCTTATGATGGGCACAATGGAAA
                              ******************************:*******************

Apricot_PaJTYG0600022743      GAGGGTACTTAAAATTATTTTGATATATATCAGTTCCAGTAGATAGTTTT
Mei_pmTOE                     GAGGGTACTTAAAATTATTTTGATATATATCAGTTCCAGTAGATAGTTTT
Mei_pmTOE^DEL                 GAGGGTACTTAAAATTATTTTGATATATATCAGTTCCAGTAGATAGTTTT
Peach_Prupe.6G091100          GAGGGTACTTCAAATTATTTTGACATATATCAGTTCCAGTAGATAGTTTT
                              **********.************ **************************

Apricot_PaJTYG0600022743      ATCTTGAGATGCCCCATCTTCAAGGTTTTCCTCAATTTTTGGATGGAAGG
Mei_pmTOE                     ATCTTGAGATGCCCCATCTTCAAGGTTTTCCTCAATTTTTTGGATGGAAG
Mei_pmTOE^DEL                 ATCTTGAGATGCCCCATCTTCAAGGTTTTCCTCAATTTTTTGGATGGAAG
Peach_Prupe.6G091100          ATCTTGAGATGCCCCATCTTCAAGGTTTTCCTCAATTTTTTGGATGGAAG
                              **************************************** *.: *.*.*

Apricot_PaJTYG0600022743      TTTTAAGGTTTCAGATGGCTTCAAACACTAATATTTCCTGTTGCCTCTCT
Mei_pmTOE                     GTTTAAGGTTTCAGATGGCTTCAAACACTAATATTTCCTGTTGCCTCTCT
Mei_pmTOE^DEL                 GTTTAAGGTTTCAGATGGCTTCAAACACTAATATTTCCTGTTGCCTCTCT
Peach_Prupe.6G091100          -------------GATGGCTTCAAACGCTAATATTTCCTGTTGCCTCTCT
                                           *************.***********************

Apricot_PaJTYG0600022743      TAATATCTCTGAAAGTAAAAGAGTACTTCTGCTGTAGTTCATTTCTTTTT
Mei_pmTOE                     TAATATCTCTGAAAGTAAAAGAGTACTTCTGCTGTAGTTCATTTGTTTTT
Mei_pmTOE^DEL                 TAATATCTCTGAAAGTAAAAGAGTACTTCTGCTGTAGTTCATTTGTTTTT
Peach_Prupe.6G091100          TAATATCTCTGAAAGTAAAAGAGTTCTTCTGCTGTAGTTCATTTGTTTTT
                              ***********************:******************** *****

Apricot_PaJTYG0600022743      GCTAGCTGCCAAAACAAAATTAATGCGTTTTAATTTTTGGAACACTTTCG
Mei_pmTOE                     GCTAGCTGCCAAAACAAAATTAATGCGTTTTAATTTTTGGAACACTTTCG
Mei_pmTOE^DEL                 GCTAGCTGCCAAAACAAAATTAATGCGTTTTAATTTTTGGAACACTTTCG
Peach_Prupe.6G091100          TCTAGCTGCCAAAACAAAATTAATGCGTTTTAATTTTTGGAACACTTTCT
                               ************************************************

Apricot_PaJTYG0600022743      CTTATTTTAGGCACATAATGATGAAAAATGTCACGCATGTCTTGGTGCTA
Mei_pmTOE                     CTTATTTTAGGCACATAATGATGAAACATGTCACGCATGTCTTGGTGCTA
Mei_pmTOE^DEL                 CTTATTTTAGGCACATAATGATGAAACATGTCACGCATGTCTTGGTGCTA
Peach_Prupe.6G091100          CTTATTTTAGGCACATAATGATGAAACATGTCACGCATGTCTTGGTGCTA
                              **************************.***********************

Apricot_PaJTYG0600022743      AAACTAAACATCCTCTCTGACTTTGCAGATGGAGCACAATGTAAATGCAA
Mei_pmTOE                     AAACTAAACATCCTCTCTGACTTTGCAGATGGAGCACAATGTAAATGCAA
Mei_pmTOE^DEL                 AAACTAAACATCCTCTCTGACTTTGCAGATGGAGCACAATGTAAATGCAA
Peach_Prupe.6G091100          AAACTAAACATCCTCTCTGACTTTGCAGATGGAGCACAATGTAAATGCAA
                              **************************************************

Apricot_PaJTYG0600022743      CGATGAGTGATCCACCTTTCAAAGGGCTAGTAATGACATCACAGCACCCA
```

```
Mei_pmTOE                      CGATGAGCGATCCACCTTTCAAAGGGCTAGTAATGACATCACAGCACCCA
Mei_pmTOE^DEL                  CGATGAGCGATCCACCTTTCAAAGGGCTAGTAATGACATCACAGCACCCA
Peach_Prupe.6G091100           CGATGAGTGATCCACCTTTCAAAGGGCTAGTAATGACATCACAGCACCCA
                               ******* ******************************************

Apricot_PaJTYG0600022743       CCATTGTGGAATGGTGTATATCCTAGTTACTTTTCCAATCAGGTGAATTT
Mei_pmTOE                      CCATTGTGGAATGGTGTATATCCTAGTTACTTTTCCAATCAGGTGAATTT
Mei_pmTOE^DEL                  CCATTGTGGAATGGTGTATATCCTAGTTACTTTTCCAATCAGGTGAATTT
Peach_Prupe.6G091100           CCATTGTGGAATGGTGTATATCCTAGTTGCTTTTCCAATCAGGTGAATTT
                               ****************************.*********************

Apricot_PaJTYG0600022743       AAATGCTTTGTCTATAAATGATATAACTTGAGTTTAATTGTATTCATTAT
Mei_pmTOE                      AAATGCTTTGTCTATAAATGATATAACTTAAGTTTAATTGTATTCATTAT
Mei_pmTOE^DEL                  AAATGCTTTGTCTATAAATGATATAACTTACGTTTAATTGTATTCATTAT
Peach_Prupe.6G091100           AAATGCTTTGTCTATAAATGATATAACTTAAGTTTAATTGTATTCATTAT
                               *****************************..*******************

Apricot_PaJTYG0600022743       ATGACTTTGTTTTATTCCTCTGACCATAACGGTACCATTTTAGCAATAAC
Mei_pmTOE                      ATGACTTTGTTTTATTTCTCTGACCATAACGGTACCATTTTTGCAATAAC
Mei_pmTOE^DEL                  ATGACTTTGTTTTATTCCTCTGACCATAATGGTACCATTTTTGCAATAAC
Peach_Prupe.6G091100           ATGACTTTGTTTTATTCCTCAGACCATAACGGTACCATTTTTGCAATAAC
                               **************** ***:******** ***********:********

Apricot_PaJTYG0600022743       CATCATAGCTCGACTTATCTGAAAAATCCCATATTGGTAACTGAATATAA
Mei_pmTOE                      CATCATAGCTCGACTTATCTGAAAAATCCCATATTGGTAACTGAATATAA
Mei_pmTOE^DEL                  CATCATAGCTCGACTTATCTGAAAAATCCCATATTGGTAACTGAATATAA
Peach_Prupe.6G091100           CATCATAGCTCGACTTATCTGAAAAATCCCATATTGGTAACTGAATATGA
                               ************************************************.*

Apricot_PaJTYG0600022743       TTAATGAAGTTGCCCCATAACCTAACTTTGTAACTAAATTTAACTCAGGT
Mei_pmTOE                      TTAATGAAGTTGCCACATAACCTAACTTTGTAACTAAATTTAACTCAGGT
Mei_pmTOE^DEL                  TTAATGAAGTTGCCACATAACCTAACTTTGTAACTAAATTTAACTCAGGT
Peach_Prupe.6G091100           TTAATGAAGTTGCCACTTAACCTAAATTTGTAACTAAATTTAACTCAGGT
                               **************.*:********.************************

Apricot_PaJTYG0600022743       TAGGTGCAAACTGAATAGGTTGGGTTGGAAGACTTTGTATTCTGTTTTCT
Mei_pmTOE                      TAGGTGCAAACTGTATAGGTTGGGTTGGGAGACTTTGTATTCTGTTTTCT
Mei_pmTOE^DEL                  TAGGTGCAAACTGTATAGGTTGGGTTGGGAGACTTTGTATTCTGTTTTCT
Peach_Prupe.6G091100           TAGGTGCAAACTGAATAGGTTGGGTTGGGAGACTTTGTATTCTGTTTTCT
                               *************:**************.*********************

Apricot_PaJTYG0600022743       ATATTGTCAATTGTGGATAGTAGATGTTGGGAAAACAAGAAGTTATAAAA
Mei_pmTOE                      ATGTTGTCAATTGTGGATAGTAGATGTTGGGAAAACAAGAAGTTATAAAA
Mei_pmTOE^DEL                  ATGTTGTCAATTGTGGATAGTAGATGTTGGGAAAACAAGAAGTTATAAAG
Peach_Prupe.6G091100           ATATTTTCAATTGTGGATAGTAGATGTTGGGAAAACAAGAAGTTATAAAA
                               **.** *******************************************.

Apricot_PaJTYG0600022743       GTAACGTTTAATAACGTGGTAAGACTTGGTCCTTTTTTTAAGTGCTTGTA
Mei_pmTOE                      GTAACGTTTAATAATGTGGTAAGACTTGGTCCTTTTTTAAG-TGCTTGTA
Mei_pmTOE^DEL                  -TAACGTTTAATAATGTGGTAAGACTTGGTCCTTTTTTAAG-TGCTTGTA
Peach_Prupe.6G091100           GTAACGTTTAATAACGTGGTAAGACTTGGTCCTTTTTTAAG-TGCTTGTA
                                ************* ***********************:*. ********

Apricot_PaJTYG0600022743       GTTTCCATAGTTTATACACTATCAAAAACTTCTTATGACTTTAAACACTG
Mei_pmTOE                      GTTTTCGTAGTTTATACACTATCAAAAACTTCTTATGACTTTAAACACTG
Mei_pmTOE^DEL                  GTTTTCGTAGTTTATACACTATCAAAAACTTCTTATGACTTTAAACACTG
Peach_Prupe.6G091100           GTTTTCGTGGTTTATACACTATCAAAAACTTCTTATGACTTTAAACACTG
                               **** *.*.*****************************************

Apricot_PaJTYG0600022743       AACTGTTGAACTGTCGCTGCTCCTATAGCGTAGTCTCTGTATTATACAAT
Mei_pmTOE                      TACTGTTGAACTGTCGCTGCTCCTATAGCGTAGTCTCTGTATTATACAAT
Mei_pmTOE^DEL                  TACTGTTGAACTGTCGCTGCTCCTATAGCGTAGTCTCTGTATTATACAAT
Peach_Prupe.6G091100           AACTGTTGAACTGTCGCTGCTCCTATAGCGTAGTCTCTGTATTATACAAT
                               :*************************************************

Apricot_PaJTYG0600022743       ATGGTCCTATTTGCTTCTGTAATCGAATCAAACTATATTTCATGTGAAGA
Mei_pmTOE                      ATGGTCCTATTTGCTTCTGTAATCGAATCAAACTATATTTCATGTGAAGA
Mei_pmTOE^DEL                  ATGGTCCTATTTGCTTCTGTAATCGAATCAAACTATATTTCATGTGAAGA
Peach_Prupe.6G091100           ATGGTCCTATTTGCTTCTGTAATCGAATCAATTTT---------------
                               *******************************: *:

Apricot_PaJTYG0600022743       ATGATCTATTTTTATTTTTCTTTCCAATTTAGCTTTCATTTAAGGTATGA
Mei_pmTOE                      ATGATCTATTTTTATTTTTCTTTCCAATTTAGCTTTCATTTAAGGTATGA
Mei_pmTOE^DEL                  ATGATCTATTTTTATTTTTCTTTCCAATTTAGCTTTCATTTAAGGTATGA
Peach_Prupe.6G091100           --------TTTTTTTTTCTTTTCCCAATTTAGCTTTCATTTAAGGTATGA
                                       *****:*** * ** ***************************

Apricot_PaJTYG0600022743       GAGGACGTTGCATTTCACACTCAGATGAAGACCTTGATTGTGTAAAATAC
Mei_pmTOE                      GAGGACGTTGCATTTCACACTTGGATGAAGACCTTGATTGTGTAAAATAC
```

```
Mei_pmTOE^DEL                   GAGGACATTGCATTTCACACTCGGATGAAGACCTTGATTGTGTAAAATAC
Peach_Prupe.6G091100            GAGGAAGTTGCATTTCACACTCGGATGAAGACCTTGATTGTGTAAAATAC
                                *****..************** .***************************

Apricot_PaJTYG0600022743        AAGTCTTCAAACTAATAGTTTACTCTTGAATCATTTGACAGGAAAGAGAA
Mei_pmTOE                       AAGTCTTGAAACTAATAGTTTACTCTTGAATCATTTGACAGGAAAGAGCA
Mei_pmTOE^DEL                   AAGTCTTGAAACTAATAGTTTACTCTTGAATCATTTGACAGGAAAGAGCA
Peach_Prupe.6G091100            AAGTCTTCAAACTAATAGTTTACTCTTGAATCATTTGACAGGAAAGAGCA
                                ******* ****************************************.*

Apricot_PaJTYG0600022743        ACAGAGAAGAGAATTGCATTAGGATCTCAAGGACCCCCCAACTGGGCTTG
Mei_pmTOE                       ACAGAGAAGAGAATTGCATTAGGATCTCAAGGACCCCCCAACTGGGCTTG
Mei_pmTOE^DEL                   ACAGAGAAGAGAATTGCATTAGGATCTCAAGGACCCCCCAACTGGGCTTG
Peach_Prupe.6G091100            ACAGAGAAGAGAATTGCATTAGGATCTCAAGGACCCCCCAACTGGGCTTG
                                **************************************************

Apricot_PaJTYG0600022743        GCAAATGCATGGCCAGGTCAGTGCTACCCCAATGCCACTGTTCTCTACTG
Mei_pmTOE                       GCAAATGCATGGCCAGGTCAGTGCTACCCCAATGCCACTGTTCTCTACTG
Mei_pmTOE^DEL                   GCAA----------------------------------------------
Peach_Prupe.6G091100            GCAAATGCATGGCCAGGTCAGCGCTACCCCAATGCCACTGTTCTCTACTG
                                ****

Apricot_PaJTYG0600022743        CAGCATCATCAGGATTCTCATTTTCAGCTACCGCTCCCTCCGCTGCTGTC
Mei_pmTOE                       CAGCATCATCAGGATTCTCATTTTCAGCTACCGCTCCCTCCGCTGCTGTC
Mei_pmTOE^DEL                   ---CATCATCAGGATTCTCATTTTCAGCTACCGCTCCCTCCGCTGCTGTC
Peach_Prupe.6G091100            CAGCATCATCAGGATTCTCATTTTCAGCTACCACTCCCCCCGCTGCTGTC
                                   *****************************.***** ***********

Apricot_PaJTYG0600022743        CACCCCTTGCAACCCTCAACCCCAACAGCCCTCAATCTCTGTTTTACTTC
Mei_pmTOE                       CACCCCTTGCAACCCTCAACCCCAACAGCCCTCAATCTCTGTTTTACTTC
Mei_pmTOE^DEL                   CACCCCTTGCAACCCTCAACCCCAACAGCCCTCAATCTCTGTTTTACTTC
Peach_Prupe.6G091100            CACCCCTTGCAACCCTCAACCCCAACAGCCCTCAATCTCTGTTTTACTTC
                                **************************************************

Apricot_PaJTYG0600022743        GCCAGCCACGGCTGCCGCCAATACTTCTCAATAGTAAAGTATCACCGAGG
Mei_pmTOE                       GCCAGCCACGGCTGCCGCCAATACTTCTCAATAGTAGAGTATCACCGAGG
Mei_pmTOE^DEL                   GCCAGCCACGGCTGCCGCCAATACTTCTCAATAGTAGAGTATCACCGAGG
Peach_Prupe.6G091100            GCCAGCCACGGCTGCCGCCAATACTTCTCAATAGTAGAGTATCACCTAGC
                                *************************************.******** ** 

Apricot_PaJTYG0600022743        GAAGGCCACACGGCCTAAATTTTCCTCCGGATTGAAGGAGCAACCATAAT
Mei_pmTOE                       GAAGGCCGCACGGCCTAAATTTTCCTCCAGATTGAAGGAGCAACCATAAT
Mei_pmTOE^DEL                   GAAGGCCGCACGGCCTAAATTTTCCTCCAGATTGAAGGAGCAACCATAAT
Peach_Prupe.6G091100            GAAAGCCGGGCGGCCAAAATTTTCCTCCAGATTGAAGGAGCAACCATAAC
                                ***.***. .*****:************.******************** 

Apricot_PaJTYG0600022743        AACGGTGGTCTTCAGATTCTTTGTATGATTCAATTAGAGATTGATATATA
Mei_pmTOE                       AACGGTGGTCTTCAGATTCTTTGTATCATTCAATTAGAGATTGATCTATA
Mei_pmTOE^DEL                   AACGGTGGTCTTCAGATTCTTTGTATGATTCAATTAGAGATTGATATATA
Peach_Prupe.6G091100            AACGGTGGTCTTCAGATTCTTTGTATGATTCAATTAGAGATTGATATATA
                                ************************** *****************.*****

Apricot_PaJTYG0600022743        CACTACAAGTTGATTTCTGACTCCTTTTCCTCTCATTACCGCACTGTTGG
Mei_pmTOE                       CACTACAACTTGATTTCTGACTCCTTTTCCTCTCATTACC--ACTGTTGG
Mei_pmTOE^DEL                   CACTACAACTTGATTTCTGACTCCTTTTCCTCTCATTACC--ACTGTTGG
Peach_Prupe.6G091100            CACTACAACTTGATTTCTGACTCCTTTTCCTCTCATTACCACTCTACTGG
                                ******** ******************************   :**. ***

Apricot_PaJTYG0600022743        ATATATCAGTACCTTTAAGTCTATTTGTATTTGTTCTCGGAAATATGTTA
Mei_pmTOE                       ATATATCAGTACCCTTAAGTCTATTTGTATTTGTTCTCGGAAATATGTTA
Mei_pmTOE^DEL                   ATATATCAGTACCCTTAAGTCTATTTGTATTTGTTCTCGGAAATATGTTA
Peach_Prupe.6G091100            ATATATCAGTACCGTTAAGTCTATTTGTATTTGTTCTCGGAAATATGTTA
                                ************* ************************************
```

B

>pmTOE^{DEL}_PmuVar_Ch1_3490_Genomic

cacgcgtcctatcacctctctctctctctctctctctgaaagcctattatattccatgactctatgagtctcctagtttatggttatggcatcacaacacacacatattaaacgacgccgttgaggtttccattctcagcctcccatcgccgccgactatat
cagtaatgcaatttattaactcattagcttaggcaaacgccgccgttcccacttcctttctctttttttactatctccggcgatcgccgacgcacccgtttgaactctttctaaaattccaagtttgagctccgatcatgtgcttctcggtgttttgtactagtg
tctgattcggtcgatctccaccgtccgattagagccggcgatcgacggtcagctgactgagctgaaaagtctagaaccggagctgaatttagagggactaaaatttatattaagagagctgagct**ATGCTGGATCTTAATCTGAACGTC**
GTCGGTTCTGACCCAAACGACGTCGAGTCATGTGGCACCCAAATGGACGAGTCGGGGACGTCGAACTCGTCCGTGGTCAATGCCGACGCATCCAGCACCAACGACGACTCGTGCTCCA
CACGCGCCGCCAGATACGACGCCGTCACGACCTTCAACTTCGATATTCTCAAGGTCAGGGGCGGAGAAGATGAAGAAGACGATGTCGTCGTGACTAAGGAGCTGTTCCCGGTCACCG
GGGCCCTGAGCAATTGGCCCGGCCAGGGGCAGTCGTCAGCGTCGTCGTCTTTGGTGAGGAAGAACTTGATGGAGCTTGGGTTCGATCATGGCGGGTCCGGAGAGGTCAGGTTGGTTC
AACAGAAACAACAGCAACCAGCCGCACCGCCACCACAGCAACAGCAGGTGAAGAAGAGCAGAAGAGGGCCGAGGTCTCGGAGCTCTCAGTATAGAGGGGTCACCTTCTATAGAAG
AACTGGTAGATGGGAATCTCATATTTGgttagtccaattttgatttccttaatatggttctattgggtttaatgtttcgttacttggatttgcttattttaatgttgtttcttttgggttgagtttgaattgcaattatgcaaatgcatctgtttca
tatagtattaatccgatttactaattattttaatttttgctgggtgcag**GGATTGCGGGAAACAAGTGTATTTGG**gtatgtgtttatgacctttccaatttgattagcttggatttagttatagtaattattgaggcaattatttgtcatgctt
ttctatttggaaagtttttgatgaaaatttaagtgttgggtttgacatag**GTGGATTTGACACTGCTCATGCTGCGGCTAG**gtaaaaagccttcttcatattttttgcttggatttcgtactgtttgtgaatttagataattagtcattgtg
gttactactgtatgtatttttattagtattcttaaattgggtttgctgaatttag**AGCCTACGATCGAGCTGCTATTAAGTTCAGAGGAGTTGATGCTGATATCAATTACAACCTCAGTGATTATGAGGAGGAT**
TTGAAACAGgtaggataatgcacttgtttcttgatgaacctatgtctgcttaatttcatatgctgagtggaatcatattttgatctatttgtaatgaatgagcag**ATGAAGAATTTGACCAAGGAAGAATTTGTGCACATACTA**
CGGAGGCAGAGCACTGGTTTCTCGAGGGGGAGCTCGAGATATAGAGGGGTTACGCTGCACAAATGTGGCCGATGGGAAGCTCGAATGGGGCAGTTCCTTGGCAAAAAGTgaggaatta
ttttatggaattttgaatttcttacagtaggtttcggtttagactttatatgatcagaggttgtcaatttgaactgagaattaatttcggattaattctcaatcagtcgttgtcttctcttttccttgtaaaaaaaaaaaaaagtccaagtgcctatttcaaa
gaaaaaaaattgtggggacggtcgtttcatcatggaggttacaatgcttgcatgcaggt**ATATATATCTTGGGCTATTCGACAGTGAAGTAGAAGCTGCAAGG**tcctaatgtcatgaattacactctacctgactgaatttatt
accctgaattctcaacctcattaggcttccatccaacacatctctccctttgatttttctggaaattagg**GCTTATGACAAGGCAGCAATCAAATGTAATGGAAGGGAAGCAGTCACCAACTTTGAGCCAAGCACA**
TATGAAGGGGAGATGATATCTGAGGCTGGTAATGAAGgtacgatttcacaaatatcactatctcttgaggtagttcaaatgcatctcctgttttcaaggcaggtggtttactaattagaaaattttcaagaaatttgtcttcgtaga
attgtgtttatatattttttttctccctgatgcag**ATGGCGATCACAATCTTGATCTGAATTTGGGGATATCTCCCCCTTCATTTGGCAATTGTCAAAAGGAAGTCGAGGGGCATCTTCAATTCCATTCCG**
GCCCTTATGATGGGCACAATGGAAAGAGGgtacttaaaattattttgatatatatcagttccagtagatagttttatcttgagatgccccatcttcaaggttttcctcaattttttggatggaaggtttaaggtttcagatggcttcaaac
actaatatttcctgttgcctctcttaatatctctgaaagtaaaagagtacttctgctgtagttcatttgtttttgctagctgccaaaacaaaattaatgcgttttaatttttggaacactttcgcttattttaggcacataatgatgaaacatgtcacgcatg
tcttggtgctaaaactaaacatcctctctgactttgcag**ATGGAGCACAATGTAAATGCAACGATGAGCGATCCACCTTTCAAAGGGCTAGTAATGACATCACAGCACCCACCATTGTGGAATGGTGT**
ATATCCTAGTTACTTTTCCAATCAGgtgaatttaaatgctttgtctataaatgatataacttacgtttaattgtattcattatatgactttgttttattcctctgaccataatggtaccatttttgcaataaccatcatagctcgacttatctgaa
aaatcccatattggtaactgaatataattaatgaagttgccacataacctaactttgtaactaaatttaactcaggttaggtgcaaactgtataggttgggttgggagactttgtattctgttttctatgttgtcaattgtggatagtagatgttgggaaa
acaagaagttataaagtaacgtttaataatgtggtaagacttggtcctttttttaagtgcttgtagttttcgtagtttatacactatcaaaaacttcttatgactttaaacactgtactgttgaactgtcgctgctcctatagcgtagtctctgtattatacaa
tatggtcctatttgcttctgtaatcgaatcaaactatatttcatgtgaagaatgatctatttttatttttctttccaatttagctttcatttaaggtatgagaggacattgcatttcacactcggatgaagaccttgattgtgtaaaatacaagtcttgaaac
taatagtttactcttgaatcatttgacag**GAAAGAGCAACAGAGAAGAGAATTGCATTAGGATCTCAAGGACCCCCCAACTGGGCTTGGCAACATCATCAGGATTCTCATTTTCAGCTACCGCTCC**
CTCCGCTGCTGTCCACCCCTTGCAACCCTCAACCCCAACAGCCCTCAATCTCTGTTTTACTTCGCCAGCCACGGCTGCCGCCAATACTTCTCAATAGTAGAGTATCACCGAGGGAAGGCC
GCACGGCCTAAattttcctccagattgaaggagcaaccataataacggtggtcttcagattctttgtatgattcaattagagattgatatatacactacaacttgatttctgactccttttcctctcattaccactgttggatatatcagtaccctta
agtctatttgtatttgttctcggaaatatgttatatctaacttgatctctaatgtaaagtatcaagtggtaatggtgggatccatctatctatccaaactactcgttcagaaagagatgttaatagtgagctcttttaagattgttatagatcgaaggtttg
tctattaacg

>pmTOE^{DEL}_cDNA

gaaagcctattatattccatgactctatgagtctcctagtttatggttatggcatcacaacacacacatattaaacgacgccgttgaggtttccattctcagcctcccatcgccgccgactatatcagtaatgcaatttattaactcattagcttaggca
aacgccgccgttcccacttcctttctctttttttactatctccggcgatcgccgacgcacccgtttgaactctttctaaaattccaagtttgagctccgatcatgtgcttctcggtgttttgtactagtgtctgattcggtcgatctccaccgtccgattagagc
cggcgatcgacggtcagctgactgagctgaaaagtctagaaccggagctgaatttagagggactaaaatttatattaagagagctgagct**ATGCTGGATCTTAATCTGAACGTCGTCGGTTCTGACCCAAACGACGTCGA**
GTCATGTGGCACCCAAATGGACGAGTCGGGGACGTCGAACTCGTCCGTGGTCAATGCCGACGCATCCAGCACCAACGACGACTCGTGCTCCACACGCGCCGCCAGATACGACGCCGTC
ACGACCTTCAACTTCGATATTCTCAAGGTCAGGGGCGGAGAAGATGAAGAAGACGATGTCGTCGTGACTAAGGAGCTGTTCCCGGTCACCGGGGCCCTGAGCAATTGGCCCGGCCAG
GGGCAGTCGTCAGCGTCGTCGTCTTTGGTGAGGAAGAACTTGATGGAGCTTGGGTTCGATCATGGCGGGTCCGGAGAGGTCAGGTTGGTTCAACAGAAACAACAGCAACCAGCCGC
ACCGCCACCACAGCAACAGCAGGTGAAGAAGAGCAGAAGAGGGCCGAGGTCTCGGAGCTCTCAGTATAGAGGGGTCACCTTCTATAGAAGAACTGGTAGATGGGAATCTCATATTT
GGGATTGCGGGAAACAAGTGTATTTGGGTGGATTTGACACTGCTCATGCTGCGGCTAGAGCCTACGATCGAGCTGCTATTAAGTTCAGAGGAGTTGATGCTGATATCAATTACAACCT
CAGTGATTATGAGGAGGATTTGAAACAGATGAAGAATTTGACCAAGGAAGAATTTGTGCACATACTACGGAGGCAGAGCACTGGTTTCTCGAGGGGGAGCTCGAGATATAGAGGG
GTTACGCTGCACAAATGTGGCCGATGGGAAGCTCGAATGGGGCAGTTCCTTGGCAAAAAGTATATATATCTTGGGCTATTCGACAGTGAAGTAGAAGCTGCAAGGGCTTATGACAAG
GCAGCAATCAAATGTAATGGAAGGGAAGCAGTCACCAACTTTGAGCCAAGCACATATGAAGGGGAGATGATATCTGAGGCTGGTAATGAAGATGGCGATCACAATCTTGATCTGAA
TTTGGGGATATCTCCCCCTTCATTTGGCAATTGTCAAAAGGAAGTCGAGGGGCATCTTCAATTCCATTCCGGCCCTTATGATGGGCACAATGGAAAGAGGATGGAGCACAATGTAAAT
GCAACGATGAGCGATCCACCTTTCAAAGGGCTAGTAATGACATCACAGCACCCACCATTGTGGAATGGTGTATATCCTAGTTACTTTTCCAATCAGGAAAGAGCAACAGAGAAGAGA
ATTGCATTAGGATCTCAAGGACCCCCCAACTGGGCTTGGCAACATCATCAGGATTCTCATTTTCAGCTACCGCTCCCTCCGCTGCTGTCCACCCCTTGCAACCCTCAACCCCAACAGCCCT
CAATCTCTGTTTTACTTCGCCAGCCACGGCTGCCGCCAATACTTCTCAATAGTAGAGTATCACCGAGGGAAGGCCGCACGGCCTAAattttcctccagattgaaggagcaaccataataacggtggtctt
cagattctttgtatgattcaattagagattgatatatacactacaacttgatttctgactccttttcctctcattaccactgttggatatatcagtacccttaagtctatttgtatttgttctcggaaatatgttatatctaacttgatctctaatgtaaagtat
caagtggtaatggtgggatccatctatctatccaaactactcgttcagaaagagatgttaatagtgagctcttttaagattgttatagatcgaaggtttgtctattaacg

>pmTOE^{DEL}_protein

MLDLNLNVVGSDPNDVESCGTQMDESGTSNSSVVNADASSTNDDSCSTRAARYDAVTTFNFDILKVRGGEDEEDDVVVTKELFPVTGALSNWPGQGQSSASSSLVRKNLMELGFDHGGS
GEVRLVQQKQQQPAAPPPQQQQVKKSRRGPRSRSSQYRGVTFYRRTGRWESHIWDCGKQVYLGGFDTAHAAARAYDRAAIKFRGVDADINYNLSDYEEDLKQMKNLTKEEFVHILRRQS
TGFSRGSSRYRGVTLHKCGRWEARMGQFLGKKYIYLGLFDSEVEAARAYDKAAIKCNGREAVTNFEPSTYEGEMISEAGNEDGDHNLDLNLGISPPSFGNCQKEVEGHLQFHSGPYDGHNG
KRMEHNVNATMSDPPFKGLVMTSQHPPLWNGVYPSYFSNQERATEKRIALGSQGPPNWAWQHHQDSHFQLPLPPLLSTPCNPQPQQPSISVLLRQPRLPPILLNSRVSPREGRTA

>pmTOE_NC_024126_Genomic

cacgcgtcctatcacctctctctctctctctctctctctctctgaaagcctattatattccatgactctatgagtctcctagtttatggttatggcatcacaacacacacatattaaacgacgccgttgaggtttccattctcagcctcccatcgccgccg
actatatcagtaatgcaatttattaactcattagcttaggcaaacgccgccgttcccacttcctttctctttttttactatctccggcgatcgccgacgcacccgtttgaactctttctaaaattccaagtttgagctccgatcatgtgcttctcggtgttttgt
actagtgtctgattcggtcgatctccaccgtccgattagagccggcgatcgacggtcagctgactgagctgaaaagtctagaaccggagctgaatttagagggactaaaatttatattaagagagctgagct**ATGCTGGATCTTAATCTGA**
ACGTCGTCGGTTCTGACCCAAACGACGTCGAGTCATGTGGCACCCAAATGGACGAGTCGGGGACGTCGAACTCGTCCGTGGTCAATGCCGACGCATCCAGCACCAACGACGACTCGT
GCTCCACACGCGCCGCCAGATACGACGCCGTCACGACCTTCAACTTCGATATTCTCAAGGTCAGGGGCGGAGAAGATGAAGAAGACGATGTCGTCGTGACTAAGGAGCTGTTCCCGG
TCACCGGGGCCCTGAGCAATTGGCCCGGCCAGGGGCAGTCGTCAGCGTCGTCGTCTTTGGTGAGGAAGAACTTGATGGAGCTTGGGTTCGATCATGGCGGGTCCGGAGAGGTCAGGT
TGGTTCAACAGAAACAACAGCAACCAGCCGCACCGCCACCACAGCAACAGCAGGTGAAGAAGAGCAGAAGAGGGCCGAGGTCTCGGAGCTCTCAGTATAGAGGGGTCACCTTCTAT
AGAAGAACTGGTAGATGGGAATCTCATATTTGgttagtccaattttgatttccttaatatggttctattgggtttcatgtttcgttacttggatttgcttattttaatgttgtttcttttgggttgagtttgaattgcaattatgcaaatgcat
ctgtttcatatagtattaatccgatttactaattatttttaatttttgctgggtgcag**GGATTGCGGGAAACAAGTGTATTTGG**gtatgtgtttatgacctttccaatttgattagcttggatttagttatagtaattattgaggcaattatttg
tcatgcttttctatttggaaagtttttttatgaaaatttaagtgttgggtttgacatag**GTGGATTTGACACTGCTCATGCTGCGGCTAG**gtaaaaagccttcttcatattttttttgcttggatttcgtactgtttgtgaatttagataattagt
cattgtggttactactgtatgtatttttattagtattcttaaattgggtttgctgaatttag**AGCCTACGATCGAGCTGCTATTAAGTTCAGAGGAGTTGATGCTGATATCAATTACAACCTCAGTGATTATGAGG**
AGGATTTGAAACAGgtaggataatgcacttgtttcttgatgaacctatgtctgcttaatttcatatgctgagtggaatcatattttgatctatttgtaatgaatgagcag**ATGAAGAATTTGACCAAGGAAGAATTTGTGCAC**
ATACTACGGAGGCAGAGCACTGGTTTCTCGAGGGGGAGCTCGAGATATAGAGGGGTTACGCTGCACAAATGTGGCCGATGGGAAGCTCGAATGGGGCAGTTCCTTGGCAAAAAGTg
aggaattattttatggaattttgaatttcttacagtaggtttcggtttagactttatatgatcagaggttgtcaatttgaactgagaattaatttcggattaattctcaatcagtcgttgtcttctcttttccttgtaaaaaaaaaaaaaaaaaagtcgaag
tgcctatttcaaagaaaaaaaattgtggggacggtcgtttcatcatggaggttacaatgcttgcatgcaggt**ATATATATCTTGGGCTATTCGACAGTGAAGTAGAAGCTGCAAGG**tcctaatgtcatgaattacactctacct
gactgaatttattaccctgaattctcaacctcattaggcttccatccaacacatctctcccttttgatttttctggaaattagg**GCTTATGACAAGGCAGCAATCAAATGTAATGGAAGGGAAGCAGTCACCAACTTTGAG**
CCAAGCACATATGAAGGGGAGATGATATCTGAGGCTGGTAATGAAGgtacgatttcacaaatatcactatcttttgaggcagttcaaatgcatctcctgttttcaaggcaggtggtttactaattagaaaattttcaagaaat
ttgtcttcgtagaattgtgtttatatatttttttctccctgatgcag**ATGGCGATCACAATCTTGATCTGAATTTGGGGATATCTCCCCCTTCATTTGGCAATTGTCAAAAGGAAGTCGAGGGGCATCTTCAA**
TTCCATTCCGGCCCTAATGATGGGCACAATGGAAAGAGGgtacttaaaattattttgatatatatcagttccagtagatagttttatcttgagatgccccatcttcaaggttttcctcaattttttggatggaaggtttaaggtttcag
atggcttcaaacactaatatttcctgttgcctctcttaatatctctgaaagtaaaagagtacttctgctgtagttcatttgtttttgctagctgccaaaacaaaattaatgcgttttaatttttggaacactttcgcttattttaggcacataatgatgaaac
atgtcacgcatgtcttggtgctaaaactaaacatcctctctgactttgcag**ATGGAGCACAATGTAAATGCAACGATGAGCGATCCACCTTTCAAAGGGCTAGTAATGACATCACAGCACCCACCATTGTG**
GAATGGTGTATATCCTAGTTACTTTTCCAATCAGgtgaatttaaatgctttgtctataaatgatataacttaagtttaattgtattcattatatgactttgttttatttctctgaccataacggtaccatttttgcaataaccatcatagctc
gacttatctgaaaaatcccatattggtaactgaatataattaatgaagttgccacataacctaactttgtaactaaatttaactcaggttaggtgcaaactgtataggttgggttgggagactttgtattctgttttctatgttgtcaattgtggatagta
gatgttgggaaaacaagaagttataaaagtaacgtttaataatgtggtaagacttggtcctttttttaagtgcttgtagttttcgtagtttatacactatcaaaaacttcttatgactttaaacactgtactgttgaactgtcgctgctcctatagcgtagtc
tctgtattatacaatatggtcctatttgcttctgtaatcgaatcaaactatatttcatgtgaagaatgatctatttttatttttctttccaatttagctttcatttaaggtatgagaggacgttgcatttcacacttggatgaagaccttgattgtgtaaaata
caagtcttgaaactaatagtttactcttgaatcatttgacag**GAAAGAGCAACAGAGAAGAGAATTGCATTAGGATCTCAAGGACCCCCCAACTGGGCTTGGCAAATGCATGGCCAGGTCAGTGCTAC**
CCCAATGCCACTGTTCTCTACTGCAGCATCATCAGGATTCTCATTTTCAGCTACCGCTCCCTCCGCTGCTGTCCACCCCTTGCAACCCTCAACCCCAACAGCCCTCAATCTCTGTTTTACTTC
GCCAGCCACGGCTGCCGCCAATACTTCTCAATAGtagagtatcaccgagggaaggccgcacggcctaaattttcctccagattgaaggagcaaccataataacggtggtcttcagattctttgtatcattcaattagagattgatct
atacactacaacttgatttctgactccttttcctctcattaccactgttggatatatcagtacccttaagtctatttgtatttgttctcggaaatatgttatatctaacttgatctctaatgtaaagtatcaagtggtaatggtgggatccatctatctatcca
aactactcgttcagaaagagatgttaatagtgagctcttttaagattgttatagatcgaaggtttgtttattaacg

>pmTOE_cDNA

gaaagcctattatattccatgactctatgagtctcctagtttatggttatggcatcacaacacacacatattaaacgacgccgttgaggtttccattctcagcctcccatcgccgccgactatatcagtaatgcaatttattaactcattagcttaggca
aacgccgccgttcccacttcctttctctttttttactatctccggcgatcgccgacgcacccgtttgaactctttctaaaattccaagtttgagctccgatcatgtgcttctcggtgttttgtactagtgtctgattcggtcgatctccaccgtccgattagagc
cggcgatcgacggtcagctgactgagctgaaaagtctagaaccggagctgaatttagagggactaaaatttatattaagagagctgagct**ATGCTGGATCTTAATCTGAACGTCGTCGGTTCTGACCCAAACGACGTCGA**
GTCATGTGGCACCCAAATGGACGAGTCGGGGACGTCGAACTCGTCCGTGGTCAATGCCGACGCATCCAGCACCAACGACGACTCGTGCTCCACACGCGCCGCCAGATACGACGCCGTC
ACGACCTTCAACTTCGATATTCTCAAGGTCAGGGGCGGAGAAGATGAAGAAGACGATGTCGTCGTGACTAAGGAGCTGTTCCCGGTCACCGGGGCCCTGAGCAATTGGCCCGGCCAG
GGGCAGTCGTCAGCGTCGTCGTCTTTGGTGAGGAAGAACTTGATGGAGCTTGGGTTCGATCATGGCGGGTCCGGAGAGGTCAGGTTGGTTCAACAGAAACAACAGCAACCAGCCGC
ACCGCCACCACAGCAACAGCAGGTGAAGAAGAGCAGAAGAGGGCCGAGGTCTCGGAGCTCTCAGTATAGAGGGGTCACCTTCTATAGAAGAACTGGTAGATGGGAATCTCATATTT
GGGATTGCGGGAAACAAGTGTATTTGGGTGGATTTGACACTGCTCATGCTGCGGCTAGAGCCTACGATCGAGCTGCTATTAAGTTCAGAGGAGTTGATGCTGATATCAATTACAACCT
CAGTGATTATGAGGAGGATTTGAAACAGATGAAGAATTTGACCAAGGAAGAATTTGTGCACATACTACGGAGGCAGAGCACTGGTTTCTCGAGGGGGAGCTCGAGATATAGAGGG
GTTACGCTGCACAAATGTGGCCGATGGGAAGCTCGAATGGGGCAGTTCCTTGGCAAAAAGTATATATATCTTGGGCTATTCGACAGTGAAGTAGAAGCTGCAAGGGCTTATGACAAG
GCAGCAATCAAATGTAATGGAAGGGAAGCAGTCACCAACTTTGAGCCAAGCACATATGAAGGGGAGATGATATCTGAGGCTGGTAATGAAGATGGCGATCACAATCTTGATCTGAA
TTTGGGGATATCTCCCCCTTCATTTGGCAATTGTCAAAAGGAAGTCGAGGGGCATCTTCAATTCCATTCCGGCCCTAATGATGGGCACAATGGAAAGAGGATGGAGCACAATGTAAAT
GCAACGATGAGCGATCCACCTTTCAAAGGGCTAGTAATGACATCACAGCACCCACCATTGTGGAATGGTGTATATCCTAGTTACTTTTCCAATCAGGAAAGAGCAACAGAGAAGAGA
ATTGCATTAGGATCTCAAGGACCCCCCAACTGGGCTTGGCAAATGCATGGCCAGGTCAGTGCTACCCCAATGCCACTGTTCTCTACTGCAGCATCATCAGGATTCTCATTTTCAGCTACC
GCTCCCTCCGCTGCTGTCCACCCCTTGCAACCCTCAACCCCAACAGCCCTCAATCTCTGTTTTACTTCGCCAGCCACGGCTGCCGCCAATACTTCTCAATAGtagagtatcaccgagggaaggccgc
acggcctaaattttcctccagattgaaggagcaaccataataacggtggtcttcagattctttgtatcattcaattagagattgatctatacactacaacttgatttctgactccttttcctctcattaccactgttggatatatcagtacccttaagtctat
ttgtatttgttctcggaaatatgttatatctaacttgatctctaatgtaaagtatcaagtggtaatggtgggatccatctatctatccaaactactcgttcagaaagagatgttaatagtgagctcttttaagattgttatagatcgaaggtttgtttatta
acg

>pmTOE_protein

MLDLNLNVVGSDPNDVESCGTQMDESGTSNSSVVNADASSTNDDSCSTRAARYDAVTTFNFDILKVRGGEDEEDDVVVTKELFPVTGALSNWPGQGQSSASSSLVRKNLMELGFDHGGS
GEVRLVQQKQQQPAAPPPQQQQVKKSRRGPRSRSSQYRGVTFYRRTGRWESHIWDCGKQVYLGGFDTAHAAARAYDRAAIKFRGVDADINYNLSDYEEDLKQMKNLTKEEFVHILRRQS
TGFSRGSSRYRGVTLHKCGRWEARMGQFLGKKYIYLGLFDSEVEAARAYDKAAIKCNGREAVTNFEPSTYEGEMISEAGNEDGDHNLDLNLGISPPSFGNCQKEVEGHLQFHSGPNDGHNG
KRMEHNVNATMSDPPFKGLVMTSQHPPLWNGVYPSYFSNQERATEKRIALGSQGPPNWAWQMHGQVSATPMPLFSTAASSGFSFSATAPSAAVHPLQPSTPTALNLCFTSPATAAANT
SQ

```
pmTOE^DEL_protein     MLDLNLNVVGSDPNDVESCGTQMDESGTSNSSVVNADASSTNDDSCSTRAARYDAVTTFN
pmTOE_protein         MLDLNLNVVGSDPNDVESCGTQMDESGTSNSSVVNADASSTNDDSCSTRAARYDAVTTFN
                      ************************************************************

pmTOE^DEL_protein     FDILKVRGGEDEEDDVVVTKELFPVTGALSNWPGQGQSSASSSLVRKNLMELGFDHGGSG
pmTOE_protein         FDILKVRGGEDEEDDVVVTKELFPVTGALSNWPGQGQSSASSSLVRKNLMELGFDHGGSG
                      ************************************************************

pmTOE^DEL_protein     EVRLVQQKQQQPAAPPPQQQQVKKSRRGPRSRSSQYRGVTFYRRTGRWESHIWDCGKQVY
pmTOE_protein         EVRLVQQKQQQPAAPPPQQQQVKKSRRGPRSRSSQYRGVTFYRRTGRWESHIWDCGKQVY
                      ************************************************************

pmTOE^DEL_protein     LGGFDTAHAAARAYDRAAIKFRGVDADINYNLSDYEEDLKQMKNLTKEEFVHILRRQSTG
pmTOE_protein         LGGFDTAHAAARAYDRAAIKFRGVDADINYNLSDYEEDLKQMKNLTKEEFVHILRRQSTG
                      ************************************************************

pmTOE^DEL_protein     FSRGSSRYRGVTLHKCGRWEARMGQFLGKKYIYLGLFDSEVEAARAYDKAAIKCNGREAV
pmTOE_protein         FSRGSSRYRGVTLHKCGRWEARMGQFLGKKYIYLGLFDSEVEAARAYDKAAIKCNGREAV
                      ************************************************************

pmTOE^DEL_protein     TNFEPSTYEGEMISEAGNEDGDHNLDLNLGISPPSFGNCQKEVEGHLQFHSGPYDGHNGK
pmTOE_protein         TNFEPSTYEGEMISEAGNEDGDHNLDLNLGISPPSFGNCQKEVEGHLQFHSGPNDGHNGK
                      ***************************************************** ******

pmTOE^DEL_protein     RMEHNVNATMSDPPFKGLVMTSQHPPLWNGVYPSYFSNQERATEKRIALGSQGPPNWAWQ
pmTOE_protein         RMEHNVNATMSDPPFKGLVMTSQHPPLWNGVYPSYFSNQERATEKRIALGSQGPPNWAWQ
                      ************************************************************

pmTOE^DEL_protein     HHQDSHFQLP--LPPLLSTPCNPQPQQPSISVLLRQPRLPPILLNSRVSPREGRTA
pmTOE_protein         MHGQVSATPMPLFSTAASSGFSFSATAPSAAVHPLQPSTPTALNLCFTSPATAAANTSQ
                       * :        :..  *:  . ..  ** :*   **  *. *  . .**  . :
```

C

```
                                EAR
Peach_Prupe.6G242400          MLDLNLSFVCNDVVSS--------------SDNNLLHLPATTSP----IQSSASFNSSSN
PmuVar_Chr1_1333              MLDLNLSFVCNDVVSS--------------SDNNLLHLPATTSP----IQSSASFNSSSN
Peach_Prupe.6G091100          MLDLNLNVVGSGPNDV--------------ESCGTQMDESGTSNSSVVNADASSTNDDSC
PmupmTOE                      MLDLNLNVVGSDPNDV--------------ESCGTQMDESGTSNSSVVNADASSTNDDSC
Peach_Prupe.2G220100          MLDLNVNITLTSDASFDYEKTKDMEVEELPQGSRTQMEDSGTSNSSVVNAEEAPTPSNAG
PmuVar_Chr5_2600              MLDLNVNITLTTDASFDYEKAKDMEVEELPQGSRTQMEDSGTSNSSVVNAEEAPTPSNAG
                              *****:... .   .               ..       : **       . :.   ..:

Peach_Prupe.6G242400          LTATGDDED---LNFLSPNDV-----------AADDNAHCDARTIQLFPLAQ---SVR--
PmuVar_Chr1_1333              LTAAGDDEDSSTLNFLSPNDV-----------AADDNAHCDARTIQLFPLAQ---SVL--
Peach_Prupe.6G091100          STRAARYDAVTTFNFDILKVR-----------GGEDEEDDVVVTKELFPVTG---GLSNW
PmupmTOE                      STRAARYDAVTTFNFDILKVR-----------GGEDEEDDVVVTKELFPVTG---ALSNW
Peach_Prupe.2G220100          EEDSTNNTTSSFVFDILKKDKDGLCNTTYYGGAKDQNPSLQFVTRSLFPVTGDGGGGNEA
PmuVar_Chr5_2600              EEDSTNNTTSSFIFDILKKDKDGLCNTTYYGEAKDQNPSLQFVTRSLFPVTGDGGGGKEA
                                 :        .     :             . :::      * .***::    .
                                                                                    NLS
Peach_Prupe.6G242400          ----SSSSSSSSSRKQWLGLSSNSG----LEVEPSYYAPAEQIVPLQHKVKKSRRGPRSR
PmuVar_Chr1_1333              ----SSSSSSSSSRKQWLGLSSNSG----LEVEPSYYAPAEQIVPLQQKVKKSRRGPRSR
Peach_Prupe.6G091100          PGQGQSSASSSLVRKNLMELGFDHGGTGEVRLVQQKQQQPAAPPPQQQQVKKSRRGPRSR
PmupmTOE                      PGQGQSSASSSLVRKNLMELGFDHGGSGEVRLVQQKQQQPAAPPPQQQQVKKSRRGPRSR
Peach_Prupe.2G220100          ECGLGLSSASSTARPQWLNLSFAESG-------GQAQAELRIMQQKKPQPRKSRRGPRSR
PmuVar_Chr5_2600              ECGLGLSSASSTARPQWLNLSFAESG-------GQAQAELRIMQQKKPQPRKSRRGPRSR
                                    *::**  * : : *.   .         .           : : :*********
                                                   AP2-R1
Peach_Prupe.6G242400          SSQYRGVTFYRRTGRWESHIWDCGKQVYLGGFDTAHAAARAYDRAAIKFRGTEADINFNV
PmuVar_Chr1_1333              SSQYRGVTFYRRTGRWESHIWDCGKQVYLGGFDTAHAAARAYDRAAIKFRGTEADINFNV
Peach_Prupe.6G091100          SSQYRGVTFYRRTGRWESHIWDCGKQVYLGGFDTAHAAARAYDRAAIKFRGVDADINYNL
PmupmTOE                      SSQYRGVTFYRRTGRWESHIWDCGKQVYLGGFDTAHAAARAYDRAAIKFRGVDADINYNL
Peach_Prupe.2G220100          SSQYRGVTFYRRTGRWESHIWDCGKQVYLGGFDTAHSAARAYDRAAIKFRGVDADINFTL
PmuVar_Chr5_2600              SSQYRGVTFYRRTGRWESHIWDCGKQVYLGGFDTAHSAARAYDRAAIKFRGVDADINFTL
                              ************************************:**************.:****:.:
                                                Linker                         AP2-R2
Peach_Prupe.6G242400          SDYEDDIKQMSNFTKEEFVHILRRQSTGFSRGSSKYRGVTLHKCG----RWEARMGQFLG
PmuVar_Chr1_1333              SDYEDDIKQMSNFTKEEFVHILRRQSTGFSRGSSKYRGVTLHKCG----RWEARMGQFLG
Peach_Prupe.6G091100          SDYEEDLKQMKNLTKEEFVHILRRQSTGFSRGSSRYRGVTLHKCG----RWEARMGQFLG
PmupmTOE                      SDYEEDLKQMKNLTKEEFVHILRRQSTGFSRGSSRYRGVTLHKCG----RWEARMGQFLG
Peach_Prupe.2G220100          GDYEEDMKQLGHLNKEEFVHVLRRQSTGASRGNSKYRGVALPKCGGAGGRWEARMAQFPE
PmuVar_Chr5_2600              GDYEEDMKQLGHLNKEEFVHVLRRQSTGASRGNSKYRGVALPKCGGAGGRWEARMAQFSE
                              .***:*:**: ::.******:******* ***.*:****:* ***    ******.**
                                                                                       EAR
Peach_Prupe.6G242400          KKYIYLGLFDSEIEAARAYDKAAIKCNGREAVTNFEPNSYDGQIMSEAHNGGSDKSLDLN
PmuVar_Chr1_1333              KKYVYLGLFDSEIEAARAYDKAAIKCNGREAVTNFEPNSYDGQIMSEAHNGGSDKSLDLN
Peach_Prupe.6G091100          KKYIYLGLFDSEVEAARAYDKAAIKCNGREAVTNFEPSTYEGEMISEAGNEDGDHNLDLN
PmupmTOE                      KKYIYLGLFDSEVEAARAYDKAAIKCNGREAVTNFEPSTYEGEMISEAGNEDGDHNLDLN
Peach_Prupe.2G220100          KK-----VFEKEGIKYNTGREAAAVTN------FVDPSIYEGEVVLDASIEGSGHNLDLS
PmuVar_Chr5_2600              KK-----VFEKEGIKCNAGREAAAVTN------FVDPSIYEGEVVLDASIEGSGHNLDLS
                              **     :*:.*    .:  :**   *       .:*. *:*::: :*   ...:.***.

Peach_Prupe.6G242400          LGIAPPLVSELQKNNSNLSSFPVQLGCDDIPIHMRTRNENCAPAPMRAQLSHGSMVASEE
PmuVar_Chr1_1333              LGIAPPSVSELQKNNSNLSSFPVQLGCDDIPIHMRTRNENSAPAPMRAQLSHGSMVASEE
Peach_Prupe.6G091100          LGISPPSFGNCQKEVEGHLQFHSGP----YDGHNGKRMEHNVNATMSDPPFKGLVMTSQH
PmupmTOE                      LGISPPSFGNCQKEVEGHLQFHSGP----NDGHNGKRMEHNVNATMSDPPFKGLVMTSQH
Peach_Prupe.2G220100          LGISQPSSGQKGNGNLGDFQFRYKE---------RPMVNGSAASAAVGQTPHVLTMVAKH
PmuVar_Chr5_2600              LGISQPSSGQKGNGNLGDFQFRNKE---------RPMVNGSAASAAVGQTPHVLTMLAKH
                              ***: *  .:  :   .  .*                  :  . :.      :   : ::.

Peach_Prupe.6G242400          PPIMSNINSSFFPIQMERATEKR--MDVNS--FPNWAWQLQGLNGGATPMPLFSAAASSG
PmuVar_Chr1_1333              PPIMSNINSSFFPIQVERATEKR--MDVNS--FPNWAWQLQGLNGGATPMPLFSAAASSG
Peach_Prupe.6G091100          PPLWNGVYPSCFSNQ-ERATEKR--IALGSQGPPNWAWQMHGQVS-ATPMPLFSTAASSG
PmupmTOE                      PPLWNGVYPSYFSNQ-ERATEKR--IALGSQGPPNWAWQMHGQVS-ATPMPLFSTAASSG
Peach_Prupe.2G220100          PALYSGMYPGFLQKYEEMDSDHNGAQAVSSPRYTNLAWQVHGNSHSVSPVQVFSIAASSG
PmuVar_Chr5_2600              PALYSGMYPGFLQKHEEMDSDHNRAQAVSSPRYTNWAWQVHGNSQSVSPVQVFSIAASSG
                              *.: ..: .. :    *  :::.    :.*   .* ***::*    .:*: :** *****

Peach_Prupe.6G242400          FPSSTATSPPAAVTQLHFPNTTILHHHFSPSTVTNNIPGFYCRS
PmuVar_Chr1_1333              FPSSTATSPPAAVTQLHFPNTTILHHHFSPSTVTNNIPGFYCRS
Peach_Prupe.6G091100          FSFSATTPPAAVHPLQPSTPTALNLCFTSPATAAANTSQ-----
PmupmTOE                      FSFSATAPSAAVHPLQPSTPTALNLCFTSPATAAANTSQ-----
Peach_Prupe.2G220100          FPSSMASTAPPAANYFPPNLQGSASASYNVGPLPFPTPSTM---
PmuVar_Chr5_2600              FPSSMASTAPPAANYFPPNFQG---SAYNVGRLPFPTPSTM---
                              *. * ::.....
```

D

>pmPET

gttttgtgataaattgtgtaaatttttctaacaagattgaaattaatgaatagtgggccaaatgtgtgtagtgaatttaccttccaatttaatcccttttttcttttgctccataacaaacaactctctctctctctctaacaactttattaacttcttaatttcg
gtgggtttgcatgaatgaagagactcacaaagtcggcctcactatgattctctcacgccggcgcttccagaaccaacttcaatttcaaccacatctccccctctctcgtctctctcaatccttttaacaaacacgtacaaaacaaagcagagctctcga
aatgaaaatgctttttccactctcatcattttctctcaccattttcccactgccattgctctctctgtgaattcacgtcccaaacaccgaacccaccacgccacccaaacaaagcccaaatctttacacaaaagca**ATGCTAGATCTCAACCTC**
AGCTTCGTCTGCAACGACGTCGTCTCCAGCTCCGACAACAACCTTCTTCACCTTCCCGCTACCACCTCCCCAATCCAGAGCTCAGCCAGCTTCAACTCCTCCTCCAACCTCACTGCCGCCGG
CGACGACGAAGACTCGTCCACCCTCAACTTCCTCTCCCCAAACGACGTCGCTGCAGACGACAACGCTCACTGTGATGCTCGGACCATCCAGCTCTTCCCGCTCGCGCAATCCGTACTCTC
TTCTTCTTCGTCGTCATCGTCTTCGAGAAAGCAGTGGCTGGGACTATCGTCCAATTCCGGATTGGAGGTGGAGCCGAGCTACTATGCTCCTGCGGAGCAGATCGTGCCCCTGCAGCAGA
AAGTGAAGAAGAGCCGGAGAGGACCCAGGTCTCGGAGCTCTCAGTATCGGGGCGTTACGTTCTATCGGAGAACTGGGAGATGGGAATCTCATATTTGgttagtgtttggctactgtgataacac
cattaacacactgtttggatactttgattgtggcatgaatttgagctctgtcatttcttttttgtgtag**GGATTGCGGGAAACAGGTGTACTTGG**gtaagaaaaatcaaaacccaccgcttagtccttttttttttcttttttttctttttttccttttt
tctttttctggctgattatatttttctttttgtcttttgtttattgttctgaaattttag**GAGGATTTGACACTGCCCATGCTGCAGCTAG**gtaagaaataagaatcctattgtttcatataaatgtttttttttaataaaaaaatttagtgcatgtt
tttttttcttttattttttttattttaacgtttcgggaatttgtgggtgtttggtaaacatgaacttag**GGCATATGATCGAGCTGCGATCAAGTTCCGTGGAACTGAAGCTGATATTAATTTCAATGTTAGTGATTATG**
AAGACGATATTAAGCAGgtcggttttggagtgttatgtgtacaatatggtgaagatttttcctggcattgtcttatttcgttctggggttgaatttgactgacgcag**ATGAGCAATTTTACAAAGGAAGAGTTTGTGCATATC**
CTGCGTCGCCAGAGCACCGGATTCTCTAGAGGAAGCTCAAAATACAGGGGAGTCACGTTGCACAAATGTGGCCGCTGGGAAGCTCGTATGGGCCAGTTTCTGGGCAAGAAGtaagaac
ccctttctgtttttactgtcattattttatgtgtcaatgtgtttgaatatggattttgagtattcatctatgtatgtggtctaagtgattatgataagtgaacaagggaacaatacaaagaattgtgttcagcaaggaccaaataaatagttgggacttcgt
ttttgtaaagtaaattgttcggatgaattgagaaattggttttcattggctatggtatttcattgcttgcctgcagg**TATGTATACCTGGGCCTATTTGATAGCGAGATTGAAGCTGCAAG**gtctttacgatattatataggcactg
ttttactcgatatctaaactttgagatcttactgaccatgggattcatttaagtcagtatttgttctttgaaatcag**GGCATATGACAAGGCTGCCATCAAATGCAATGGAAGAGAAGCAGTCACCAACTTTGAGCCAA**
ACTCATATGATGGTCAGATAATGTCTGAGGCCCACAATGGAGgtatattgttgatagagaatccatttttgtaagatctttgtgtgtaatcctttgtcaatttagttgagcaaaaaaatttttttttaaaaaaaaaaaaatcgaacta
ttcttttatttctaccagtacatcatctatctacaaccctcttggtacag**GCAGTGACAAAAGTCTTGATCTGAACTTGGGAATTGCTCCCCCTTCGGTTTCTGAGCTCCAAAAGAACAACAGCAATTTGAGC**
AGCTTCCCTGTTCAGCTAGGCTGCGATGATATTCCTATTCACATGAGAACAAGGgtaatgttgagatcaactttgaaagccccactcagctgaattcacctttgctattgtacagcagtcctttgcaacttaagctactcc
ctttaaagcattttattgttctgtttacag**AATGAGAACTCTGCTCCGGCACCTATGAGGGCTCAACTGTCTCATGGCTCAATGGTGGCGTCTGAGGAGCCTCCTATAATGAGCAACATAAATTCCA**
GTTTCTTTCCCATCCAAGTGgtaatttacattcacttctagtcactatcataatttattataagaagttatcaaaccttaatttttcaatgttttcatttgattgtgttattggtgtacacttcaatttttttatgttaaatcttctatccaatttacaaa
gaatctccttgttttttacattcgatattcccctattggtccttgttttcttaacccttggaagaagcattgcaggcttccctaatatgaacaaattctttgattgcaaagagggtttatttcctgtttatcaccaatcactgacaattctagttttgctgcgt
ttgttaacaattcgacttcttttgtattttcag**GAAAGAGCAACGGAAAAGAGAATGGATGTCAACTCCTTCCCAAATTGGGCATGGCAACTCCAAGGCCTTAATGGTGGAGCAACTCCAATGCCAC**
TCTTCTCTGCTGCAGCATCATCAGGATTCCCTTCTTCAACAGCCACTTCACCACCAGCTGCTGTCACTCAACTTCATTTTCCTAACACAACTATTCTCCACCACCATTTTTCACCATCGACAG
TCACCAACAACATACCCGGTTTCTATTGCAGGAGCTGAaaaccagaaccagataggatataaaccacgacacattagtattttagtgtagaatgtagtcacgggaggagtagaaaaagattggtaatgaaatttgttttgctat
gatcatgtaaagcttatgaaacttgaggatcttggtctagaggcatggtaaaattgaggtagttttgccttcttctagcttgtggtcacattcttttgcatgcctccttcctttgaggggtgctgaaaagcaggtggagcaaaatctgatttggaagctt
aaaacagttaaatttcaaatgccttttctttttactgctgaatagtttgtactttagcgtgaaatgaatggtatcttatttctgattcccagattggtagttgctcataaa

3'END of *pmPET* coding sequences

```
pmPET        CTCTTCTCTGCTGCAGCATCATCAGGATTCCCTTCTTCAACAGCCACTTCACCACCAGCT
pmPET SNP1   CTCTTCTCTGCTGAAGCATCATCAGGATTCCCTTCTTCAACAGCCACTTCACCACCAGCT
pmPET SNP2   CTCTTCTCTGCTGCAGCATCATGAGGATTCCCTTCTTCAACAGCCACTTCACCACCAGCT
             *************.******** *************************************

pmPET        GCTGTCACTCAACTTCATTTTCCTAACACAACTATTCTCCACCACCATTTTTCACCATCG
pmPET SNP1   GCTGTCACTCAACTTCATTTTCCTAACACAACTATTCTCCACCACCATTTTTCACCATCG
pmPET SNP2   GCTGTCACTCAACTTCATTTTCCTAACACAACTATTCTCCACCACCATTTTTCACCATCG
             ************************************************************

pmPET        ACAGTCACCAACAACATACCCGGTTTCTATTGCAGGAGCTGA
pmPET SNP1   ACAGTCACCAACAACATACCCGGTTTCTATTGCAGGAGCTGA
pmPET SNP2   ACAGTCACCAACAACATACCCGGTTTCTATTGCAGGAGCTGA
             ******************************************
```

Translation of 3'END of *pmPET* coding sequences

```
pmPET        LFSAAASSGFPSSTATSPPAAVTQLHFPNTTILHHHFSPSTVTNNIPGFYCRS-
pmPET SNP1   LFSAEASSGFPSSTATSPPAAVTQLHFPNTTILHHHFSPSTVTNNIPGFYCRS-
pmPET SNP2   LFSAAAS-
             **** **
```

E

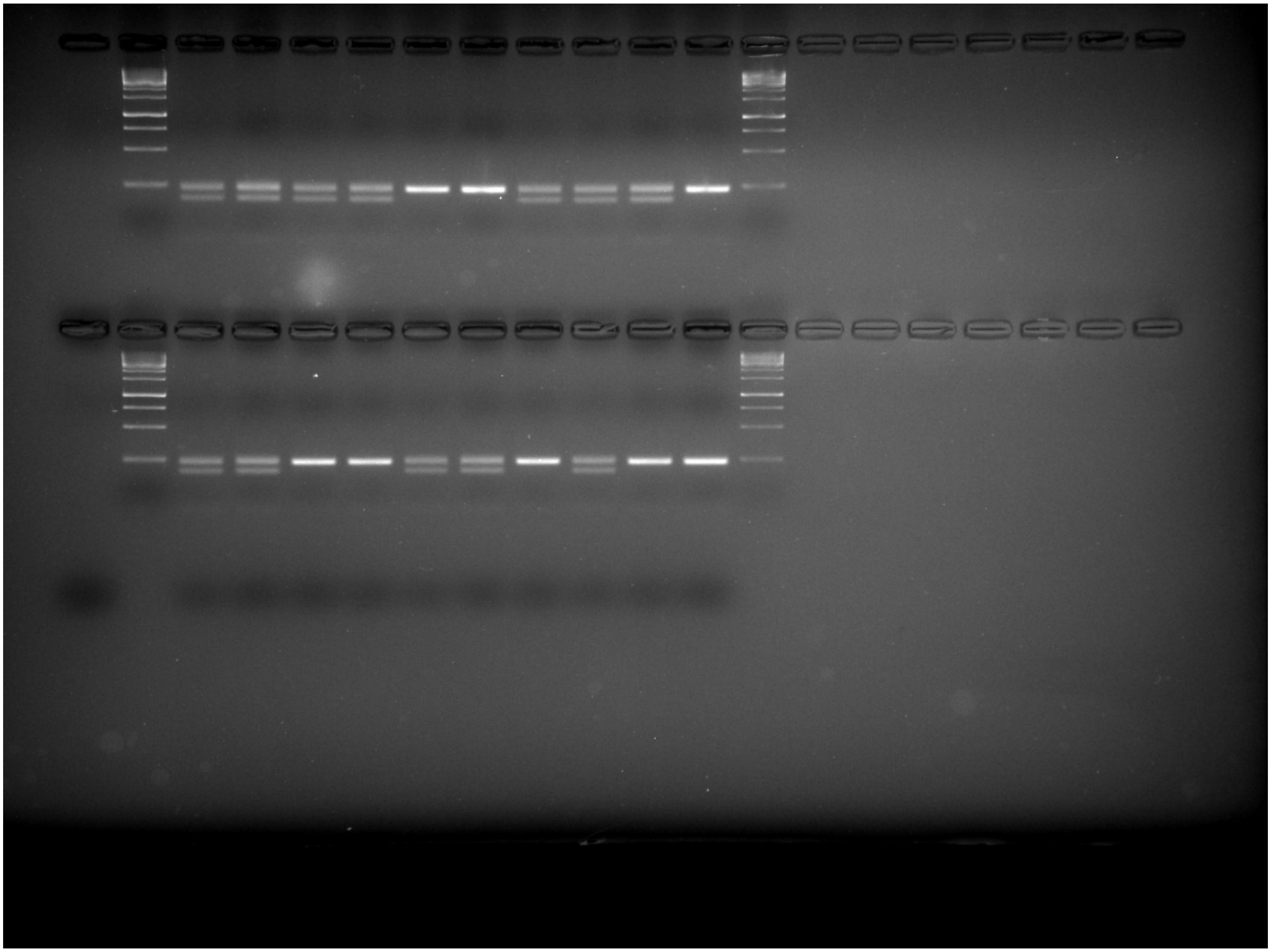

Supplement: Supplementary file 1 — Supplementary Information. [file 41598_2024_57589_MOESM1_ESM.pdf]
